# Women’s experiences of discussing health behaviours within their maternity care: a systematic review and meta-synthesis

**DOI:** 10.1186/s12978-026-02368-z

**Published:** 2026-05-29

**Authors:** Lucy Hulme, Debbie M Smith, Sarah Peters

**Affiliations:** Manchester Centre for Health Psychology, University of Manchester, Manchester, United Kingdom

**Keywords:** Pregnancy, Postpartum, Maternity care, Lived experience, Qualitative, Systematic review

## Abstract

**Introduction:**

Health behaviours are important because they have been linked to pregnancy outcomes. During maternity care women are provided with health information, but little is known about how women experience these discussions. Therefore, a systematic review was conducted to investigate women’s experiences of discussing health behaviours within their maternity care interactions.

**Methods:**

Bibliographic databases were searched to retrieve studies reporting qualitative data regarding women’s experiences. Included records were assessed for quality using the Critical Appraisal Skills Programme tool and then thematically synthesised. A professional and public stakeholder group was consulted for feedback at multiple stages.

**Findings:**

Women’s experience from 81 records was captured within three themes. Theme 1 presents how women feel they do not know enough about health, theme 2 shows that women want to have a discussion about health, and theme 3 describes how healthcare provider communication style can impact how engaged women are in a discussion.

**Conclusion:**

This suggests that healthcare providers should be engaging women in conversations where women can express motivation for change, and then together they can both contribute to shared discussions about the woman’s health. Future research should focus on providing support for healthcare providers to achieve this through training and intervention.

**Supplementary Information:**

The online version contains supplementary material available at 10.1186/s12978-026-02368-z.

## Abstract (plain language summary)

Health behaviours, such as eating healthily, exercising, smoking etc., are important because they can impact the outcome of a pregnancy. During maternity care healthcare providers talk to women about these healthy behaviours, however, we do not know how women feel during these conversations. Therefore, a systematic review took place to explore women’s experiences of talking about health behaviours within their maternity care.

In this systematic review, all previous research that looked into women’s feelings about this topic was found and then analysed. A stakeholder group, including relevant professionals and members of the public, were involved in analysing and summarising the previous research to ensure that women’s experiences were being understood and represented correctly.

It was found that there were three common feelings that women had when talking about health behaviour. Firstly, women believe they do not know enough about health behaviour, and this could lead to women feeling upset. Secondly, women want to have the opportunity to ask their healthcare provider questions. Lastly, how a healthcare provider talks to a woman can impact how women feel during the conversation, for example, if a healthcare provider talks to a woman without letting her ask questions this feel discouraging. These findings mean healthcare providers need to be talking to women about healthy behaviour in a way that lets women feel part of the conversation, this will help them build their confidence to make healthy choices.

**Supplementary Information:**

The online version contains supplementary material available at 10.1186/s12978-026-02368-z.

## Introduction

The experience of pregnancy can be defined as encompassing the 1001 days from conception until two years postpartum [1]. Healthcare institutions and policy recommend that pregnant women are provided with health advice, including information on health behaviours such as smoking cessation, alcohol consumption, exercise, and diet, but little is known about how women experience these discussions [2]. Health behaviours are actions and habits that contribute to overall health maintenance and to health improvement [3]. These four specific behaviours are important because they have been linked to adverse pregnancy outcomes and non-communicable diseases, issues which place high economic pressures on healthcare institutions [4–6]. Throughout the maternity journey, women have a unique schedule of regular contact with healthcare providers (HCP) who can help them initiate or maintain these health changes [7]. Which has benefits such as women being in a state of improved health for any future pregnancies [8], and increasing the positive health outcomes of their existing children [9].

The 1001 critical days of maternity contains multiple Teachable Moment (TM) opportunities to promote and facilitate health behaviour change [10]. The TM model states that individuals can spontaneously begin making healthier choices after experiencing a health event, such as pregnancy [11], or even as a result of healthcare interactions [12, 13]. Phelan [14] explained that the reason for spontaneous changes to behaviour were due to a mothers increased risk perception for themselves and their baby, their strong emotional response to being pregnant, and the woman’s changing self-concept and social role as a mother. However, Olander et al., [10] argues that McBride’s original model of TMs only addresses the internal constructs that motivate health behaviour change and suggested that the Capability Opportunity Motivation Behaviour (COM-B) framework, originally created by Michie et al., [15], better explains how external constructs can also influence a pregnant person’s ability to succeed in making health behaviour changes. Including the COM-B model can highlight accessibility to appropriate support as an external factor that can influence women’s health behaviour change decisions [10]. These external influences are not identified by McBride’s TM model alone [16], meaning that McBride’s TM model and Michie’s COM-B model together provides the most valid theoretical foundation to explore TMs in pregnancy [16, 17].

It is important to know how women experience health behaviour discussions throughout their maternity care because there are potentially TMs throughout the first 1001 critical days and without knowing what those TMs are or how they come to be, maternity HCPs cannot act upon them [10]. For example, some women have highlighted the postnatal period as their preferred time to make health behaviour changes because they perceive their external environment at this time as more supportive in which to make lasting changes [8, 18]. Yet HCPs that support women postnatally are missing this opportunity where women’s internal motivation is high and their external environment is facilitating the opportunity for change [19, 20]. Furthermore, it is vital that any potential TMs during maternity HCPs are identified by the women themselves or interpreted by researchers from their lived experience. The reason this is important is that a TM, as defined by McBride et al., [10] alone, is largely an internal change in motivation and consequently, women have the potential to be open to behaviour change that then health care providers can support them through. But as Lawson and Flocke [12] have established, it is difficult to confidently categorise what is in actuality a TM. Therefore, it is important for the TM theory, when considering the context of pregnancy as a unique health event, to be utilised to explore how women experience health behaviour discussions within their personal maternity care journey and attempt to better understand what interactions they feel are a TM.

HCPs aim to support women throughout the maternity journey to make health behaviour changes [7, 19]. However, it is currently unknown whether women feel supported in these discussions nor how they want to be supported in these discussions. This review aimed to investigate women’s discussion experience as an integral first step in improving how HCPs support women’s physical health across the 1001 critical days. The choice to focus on women’s experience for these discussions is purposefully broad to allow information about emotional and physical lived experience, expectation and perception of care, and responsiveness to care to be identified [21]. Therefore, the current systematic review asked the question, in the existing literature what are women’s experiences of discussing health behaviours within their maternity care interactions?

## Methodology

The review was conducted in line with the Centre for Reviews and Dissemination and reported using the Preferred Reporting Items for Systematic Reviews and Meta-Analyses statement (PRISMA) [22]. The review protocol was registered in PROSPERO [23] (CRD42022370962) on 27 October 2022.

### Stakeholder involvement

A stakeholder group of five people were invited to contribute and provide insight to this research, this included: midwives, General Practitioners (GP), and service users – this group represents the views and experiences of both HCP and service users which results in a well-rounded and meaningful array of contributions [24]. Their contributions took place during the refinement of the search strategy criterion and then during the thematic synthesis of data.

### Development of review question

The SPiDER framework for qualitative systematic syntheses [25] was used to guide the creation of the review question. In Table 1, the review question concepts have been mapped onto the corresponding framework constructs.

**Table 1:** The key points from the research question mapped on to the SPiDER framework

SPiDER constructs	Sample	Phenomenon of Interest	Design	Evaluation	Research type
Key points from the research question	Research with a sample of women currently going through perinatal care or have been through perinatal care in the past.	Health behaviour discussion between women and perinatal care provider.	Qualitative data collection that allows the participants to speak in their own words and the researcher to probe for more detail	Experience as defined by Wolf [21].	Primary research studies reporting qualitative findings within either a qualitative / mixed methodology study or qualitative analysis approach.

### Eligibility of studies

The creation of the eligibility criteria was also guided by the SPiDER framework.

The final eligibility criterion is in Table 2. These eligibility criteria were used at the title and abstract screening and again at the full text screening stage to decide which records would be included in the review analysis.

**Table 2:** Inclusion and Exclusion Criteria

Include	Research with a sample of women currently accessing pregnancy or postnatal care. Research with a sample of women who have accessed this care in the past (and are reflecting on the period of 1001 days, also known as pregnancy and 2 years postpartum). Health behaviour discussion between women and maternity care provider. Interviews. Focus groups. Examples indicators of experience: Lived experience, expectation, belief, understanding, perspective, emotion. Qualitative methods Mixed methods
Exclude	Research that aggregates a sample of women and others (e.g., women and midwives). Research where the sample of women is <18. Pregnancy specific health behaviours (e.g., breastfeeding). If a lifestyle health behaviour is conflated with other non-lifestyle health behaviours and it is not clear which behaviours are being talked about in an instance. Then exclude based on the fact the phenomenon of interest cannot be extracted in isolation. Surveys with no follow procedure up that allows researchers to probe for clarification. Non-primary research. Research interventions. Mixed methods if qualitative data cannot be extracted in isolation.

### Search strategy

The SPiDER framework was used to identify key terms for the database search strategy [25].During the refinement of the search strategy, stakeholder knowledge was utilised when defining health behaviour. After in-person and online discussions with the stakeholders, it was decided that the search strategy remain broad to allow a range of behaviours to be included. Stakeholders explained how important different health behaviours are, and how they all play an important role in maternity care and women’s lives. Therefore, health behaviour discussions as a concept were purposefully kept broad to allow a natural emergence of phenomena that women wanted to discuss with their HCP [26].

Table 3 shows each construct of the SPiDER framework, its link to the research question, and an example of some of the search terms that were created for that specific construct. The complete search strategy can be accessed in the supplementary materials.

**Table 3:** An extract of key search terms generated for each construct mapped onto the SPiDER framework

SPiDER constructs	Sample	Phenomenon of Interest	Design	Evaluation	Research type
Search terms	Pregnant women; pregnancy; postpartum women; postpartum period	Health behavio? r*; health behavio? r* change; lifestyle	Interview; focus group	Experience; expectation; perspective; understand*	Qualitative; qualitative research; mixed methods

The ? symbol in the search terms is to account for different spellings of the word. The * symbol in the search terms is to account for different tenses of the word

Searching took place from November 2022 until June 2023, and then in November 2025. The databases searched were Maternity and Infant Care Database, MIDIRS (Ovid), MEDLINE (Ovid), PsycINFO (Ovid), and the Cumulative Index to Nursing and Allied Health Literature – Plus, CINAHL-P (EBSCOhost). Search strategy terms were used in grey literature searching via Google Scholar and the Monash University grey literature library. Once database searching and grey literature searching were complete, the included records had both their reference list and cited in information searched for further relevant papers. There were no date or language restrictions for inclusion of records [27].

### Study selection

There were three reviewers in total, LH who reviewed 100% of the records, HT who reviewed a randomised sample of 5% and then 10%, and DS who acted as the tie breaker for conflicts. The initial rater-reliability check at the title and abstract stage had the second reviewer (HT) screen a randomised 5% of records, and an initial Cohen’s k of 0.59 showed moderate agreement [28]. Conflicts were resolved through discussion between the first and second reviewer until there was complete agreement. The second rater-reliability check took place at the full text review stage, where the second reviewer screened another randomised 10% and a perfect agreement was reached (Cohen’s K = 1 [28], . Grey literature and forward and backward citing were then used to search for additional records.

### Data extraction and quality assessment

A quality assessment was conducted using the Critical Appraisal Skills Programme (CASP) tool for qualitative reviews [29]. This specific assessment tool was chosen due to its methodological relevance, and due to the potential for the quality assessment output to be utilized during data analysis [30, 31]. Only records that the researchers’ asses to be relevant and rich enough in information are then analysed bottom-up, whilst records that do not meet this threshold are then only analysed top-down [31]. This means that the records with the richest and most relevant information were more impactful in shaping the final analysis.

Because the CASP tool output would be directly impacting the analysis process, a decision was made to modify the CASP tool to allow for the capture of more nuance between the records. The original binary response options of ‘Yes’ and ‘No’ was not appropriate in all situations, and so a third response option of ‘Somewhat’ was added. This new ‘Somewhat’ response encompassed records that made a reasonable attempt at fulfilling a particular CASP quality domain but had noteworthy limitations [31].

In keeping with its qualitative roots, the CASP tool does not have an established scoring system, and instead encourages the researcher to engage with the items on the checklist and define their own assessment criteria [29]. In the current review, the checklist items 8 and 9 were identified as the priority, and so being assessed as ‘Yes’ for these items indicated a high-quality record, being assessed ‘Somewhat’ indicated a medium-quality record, and being assessed ‘No’ indicated a low-quality record.

To ensure a thorough and well justified assessment of quality, two researchers (LH and HT) assessed a randomised 10% of the included studies, and had in-depth discussions around each of the individual questions used as part of the CASP tool until 100% agreement was reached [26, 32]. Data from all the records were then systematically extracted using a data extraction tool (supplementary materials) to collect demographic and health discussion experience information.

### Data Synthesis

**Fig. 1:**
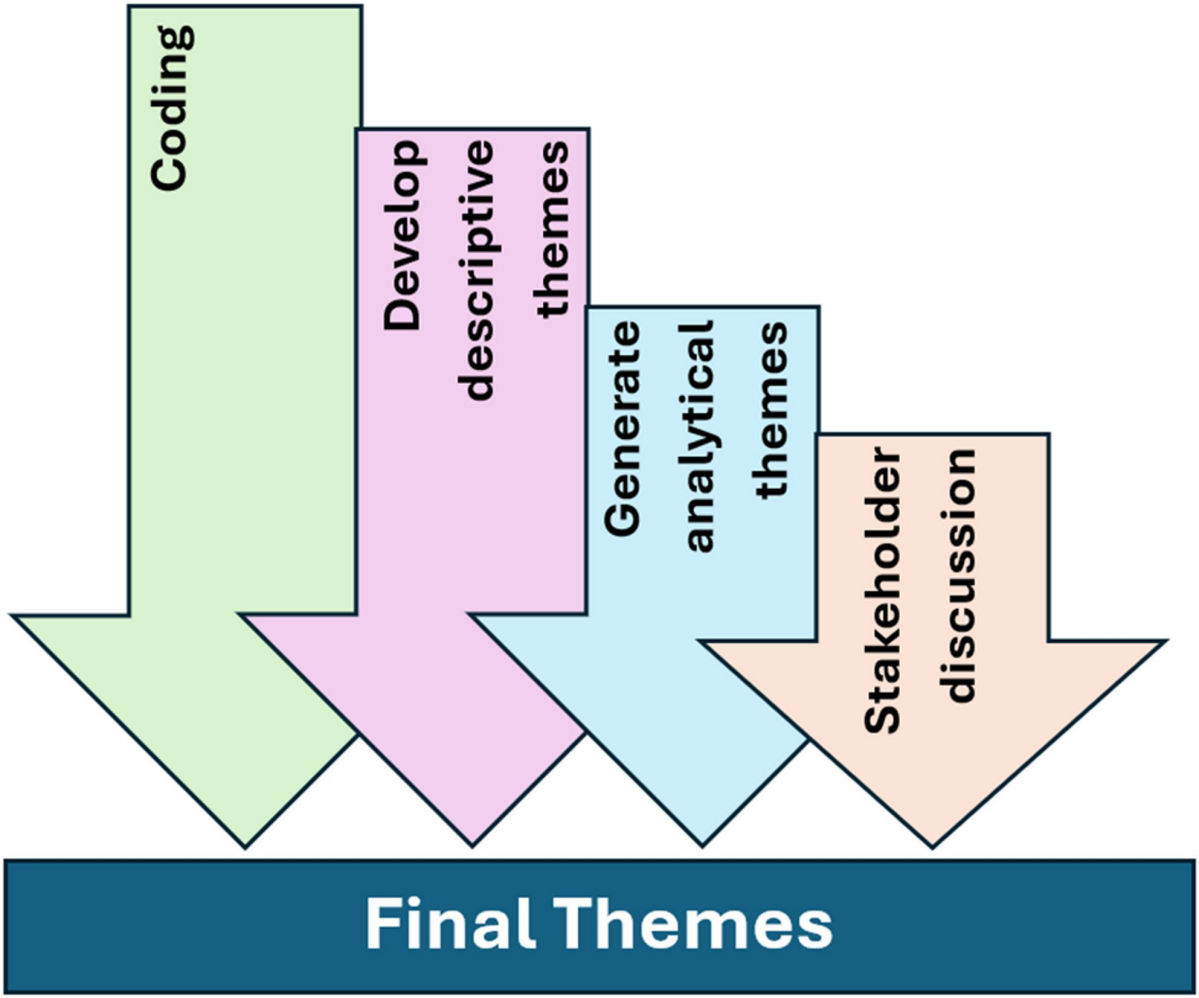
A Summary of the Adapted Thomas and Harden’s Stages of Thematic Synthesis

Data were synthesised using thematic synthesis [33]. There are three non-sequential stages (summarised in Fig. 1), and for the purposes of the current review, stages one and two have been modified as suggested by Long, French and Brooks [31] to weight higher quality studies as more impactful on the synthesis than lower quality studies.

Stage one began with line-by-line coding of the extracted data from high and medium quality records. Codes were generated inductively with a mix of semantic and latent labels [34]. During the coding process, codes were organised by similarity of concept into groups for the purpose of building up common concepts. This continuous development of descriptive themes is stage two. After all the high and medium quality records were coded to completion, the low-quality records were coded using the code labels that had already been created and added to the descriptive themes already in development at that time.

Stage three was the generation of more explicitly analytical themes. This consisted of linking the different common concepts together to create a richer and more nuanced narrative of experience. Three researchers contributed to the generation of analytical themes through continuous reflexive group discussion. Lastly, once the stakeholder feedback had been discussed and implemented the final themes were written up and formally defined.

Towards the end of data synthesis, preliminary analytical themes were presented independently to each member of the stakeholder group for feedback. Stakeholders were asked to focus on whether the findings reflected their own personal experience as either a HCP or service user and which concepts felt most important to emphasise in their opinion [35–37]. Feedback was then discussed further to ensure clarity of understanding and implemented within the final themes.

### Reflexivity

Researcher LH carried out the thematic synthesis. LH has a psychology background, but has not personally experienced maternity care and is not a HCP clinician. To ensure respectful engagement with data that focuses on mothers and HCPs, LH discussed the analysis process and then presented the preliminary findings to the stakeholder group to ensure they were in agreement with how LH had interpreted the findings. Throughout the research process, LH acknowledged their position as an outsider to both the experience of motherhood and maternity healthcare and therefore consulted with the stakeholder group whenever possible to ensure they correctly understood the experiences being represented by the data and were presenting them respectfully and accurately.

### Findings

**Fig. 2:**
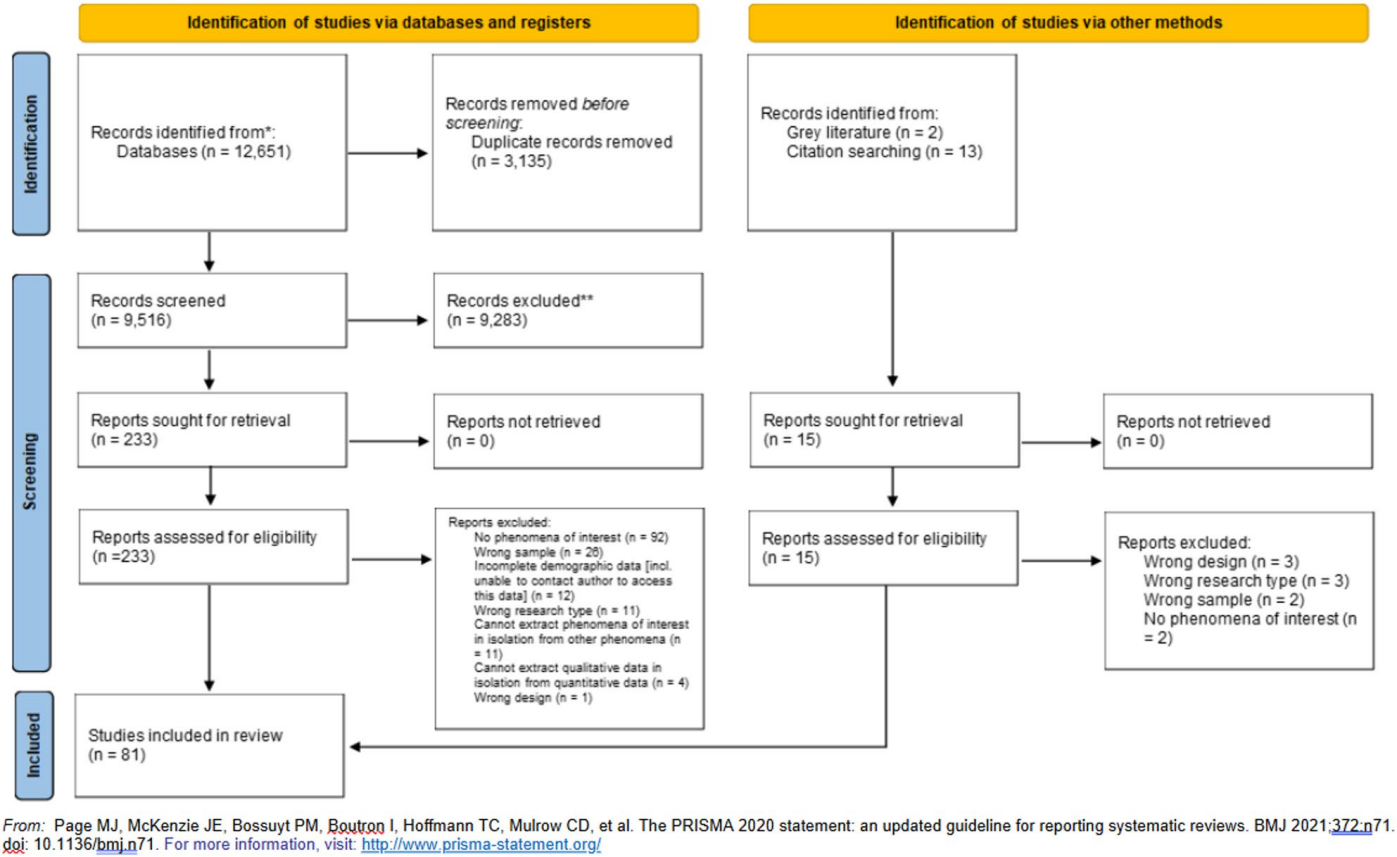
A PRISMA Flow Chart to Represent the Identification of Records Included in this Review

After removing duplicates, 9,516 records were screened for eligibility. A total of 81 records were eligible for inclusion as shown in Fig. 2. The initial stakeholder decision to keep the inclusion of health behaviours broad led to the current review including a uniquely large number of papers for a qualitative synthesis with 81 records after both rounds of screening in 2022 and 2025 – but this can still be comparable in scale to similar systematic reviews within the area of maternal health like Rockliffe, Peters [16]. Across these records the experiences of 2041 women were represented from 22 countries – further demographic information can be viewed in Table 4. One non-English paper was translated by a volunteer who was a native speaker.

**Table 4:** Demographic Information Across the Final 81 Included Records

Author/s	Study Title	Participant information				Maternity care information	
		*N*	Age in years*	Ethnicity / Nationality	Eligibility criteria	1001 Days timeframe**	Country care provided in
Arreciado Marañón et al., (2021)	Understanding factors that influence the decision to be vaccinated against influenza and pertussis in pregnancy: A qualitative study	18	33.2	Spanish; Other	Inclusion: being at least age 18; being at 27–40 weeks of pregnancy; having received the tetanus, diphtheria, pertussis vaccine; not having communication difficulties.	Pregnant	Spain
Atkinson et al., (2016)	Is pregnancy a teachable moment for diet and physical activity behaviour change? An interpretative phenomenological analysis of the experiences of women during their first pregnancy	7	28–42	White	Inclusion: pregnant for the first time.	Pregnant	England
Atkinson et al., (2017)	Unconscious collusion: An interpretative phenomenological analysis of the maternity care experiences of women with obesity (BMI ≥ 30 kg/m²)	15	21–39	N/A	Inclusion: having a BMI ≥ 30 kg/m² at their first antenatal visit (regardless of parity and type of delivery). Exclusion: experience of premature birth; stillbirth; or baby was admitted to Neonatal Unit.	Within 2 years postpartum	Republic of Ireland
Barbosa-Leiker et al., (2020)	Daily Cannabis Use During Pregnancy and Postpartum in a State With Legalized Recreational Cannabis	19	18–39	Caucasian	Exclusion: being under age 18; not pregnant or not recently postpartum (i.e., within 3 months of giving birth); using cannabis less than occasionally.	Pregnant, within two years postpartum	USA
Baron et al., (2017)	Exploring health education with midwives, as perceived by pregnant women in primary care: A qualitative study in the Netherlands	22	30.5, 23–37	Dutch; Mixed; Other	Inclusion: pregnant women under primary midwife-led care; had at least one antenatal visit; a good speaking ability of either Dutch or English.	Pregnant	Netherlands
Beckham et al., (2015)	‘‘We Know but We Don’t Really Know’’: Diet, Physical Activity and Cardiovascular Disease Prevention Knowledge and Beliefs Among Underserved Pregnant Women	50	18–36	African-American; White	Inclusion: English-speaking, self-identified as African American or Caucasian; 12 weeks gestation or more at the time of the focus group; Medicaid recipient; receiving prenatal care at a UNC-based clinic or Pitt County clinic; 18 years of age or older.	Pregnant	USA
Bianchi et al., (2016)	Concerns, attitudes, beliefs and information seeking practices with respect to nutrition-related issues: a qualitative study in French pregnant women	40	30.5	N/A	Inclusion: be pregnant; French-speaking; had not developed gestational diabetes; not experiencing a multiple pregnancy.	Pregnant	France
Bianchini et al., (2020)	Prevalence of Alcohol and Tobacco Use and Perceptions Regarding Prenatal Care among Pregnant Brazilian Women, 2017 to 2018: A Mixed Methods Study	14	25.4	White; Black; Brown; Other (Indigenous)	Inclusion: pregnant; assisted in primary care units and through the Family Health Strategy in the city of Santa Maria.	Pregnant	Brazil
Bookari et al., (2017)	Informing Nutrition Care in the Antenatal Period: Pregnant Women’s Experiences and Need for Support	26	20–29	Australian; other	Inclusion: Pregnant or had recently given birth.	Pregnant; within two years postpartum	Australia
Booth et al., (20180	A qualitative analysis of postpartum contraceptive choice	47	18–39	Black; White	Inclusion: 18 years old or older; receiving post-partum care from the affiliated hospital; English speaking.	Within two years postpartum	USA
Boydell et al., (2020)	Women’s experiences of accessing postpartum intrauterine contraception in a public maternity setting: a qualitative service evaluation	35	20–40+	White British/Scottish; White other; Other	Inclusion: received postpartum intrauterine contraception; consented to take part in the wider health service evaluation.	Within two years postpartum	Scotland
Bradford et al., (2024)	Diabetes in pregnancy: Women’s views of care in a multi-ethnic, low socioeconomic population with midwifery continuity-of-care	19	32	Māori; Pacific Peoples; Asian; South Asian; Other Asian; European & other	Inclusion: given birth within the previous 6–18 months; received their care through the Te Whatu Ora Counties Manukau DiP service; living in the Auckland region; able to give informed consent and converse in English.	Within two years postpartum	New Zealand
Bulman et al., (2013)	HIV testing in pregnancy: Using women’s voices to inform policy	12	21–35	N/A	Inclusion: be English speaking; pregnant; and of legal age.	Pregnant	Canada
Chana & Haith-Cooper (2019)	Diet and physical activity in pregnancy: a study exploring women’s beliefs and behaviours	12	22–36	British; Pakistani; Polish	Inclusion: aged over 16 years with an ongoing, low-risk pregnancy; a good understanding of written and verbal English. Exclusion: having a medical condition.	Pregnant	England
Congdon et al., (2020)	Meeting the Needs of Postpartum Women With and Without a Recent Preterm Birth: Perceptions of Maternal Family Planning in Pediatrics	41	30.1	Latina; White; Asian; African-American; Pacific Islander; Other	Inclusion: English or Spanish-speaking with infants 12 months or younger who were getting care from a paediatrician or paediatric nurse practitioner; only women who delivered before 37 weeks of gestation at intensive care nursery and high-risk infant follow up sites. Exclusion: women with term infants (> 37 weeks of gestation at birth) at intensive care nursery and high-risk infant follow up sites.	Within two years postpartum	USA
Cronin et al., (2023)	Facilitators and barriers to attending postpartum screening in women with a recent pregnancy complicated by gestational diabetes mellitus: a qualitative study	27	34	N/A	Inclusion: gave birth at University Maternity Hospital Limerick between April 2020 and April 2022; a diagnosis of GDM confirmed by an OGTT in accordance with the IADPSG diagnostic criteria.	Within two years postpartum	Republic of Ireland
Cunningham et al., (2018)	Communication with health professionals: The views of pregnant women with a raised BMI	9	19–38	White British	Inclusion: have a raised BMI (30 kg/m2 or more); be pregnant at the time of study recruitment. Exclusion: did not have a good understanding of English.	Pregnant	England
Dinsdale et al., (2016)	“As soon as you’ve had the baby that’s it…” a qualitative study of 24 postnatal women on their experience of maternal obesity care pathways	24	20–42	N/A	Exclusion: had experienced adverse pregnancy outcomes (miscarriage, late fetal loss or stillbirth); under 16 years of age at the time of booking; could not speak or read English.	Pregnant	UK
Draffin et al., (2016)	Exploring the needs, concerns and knowledge of women diagnosed with gestational diabetes: A qualitative study	19	34.5, 29–43	White; Black African; Pakistani; Latin American; Bangladeshi; Indian	Inclusion: aged 18–45 years; currently pregnant with GDM or with a history of GDM in a recent pregnancy (up to 12 months post-natal). Exclusion: unable to adequately understand verbal explanations in English; had special communication needs.	Pregnant; within two years postpartum	UK
English & Greyson (2022)	“You still have that fear”: Policy constraints on informed decision making about legalized cannabis use during pregnancy and lactation	23	21–39	N/A	Inclusion: currently or recently (within 12 months) pregnant; had considered or used cannabis during pregnancy or lactation; living in Massachusetts; 21–45 years old; female; interests in parenting, pregnancy, or breastfeeding.	Pregnant; within two years postpartum	USA
Fernández-Sola et al., (2018)	Sexuality throughout all the stages of pregnancy: Experiences of expectant mothers	15	27–38	N/A	Inclusion: being pregnant; maintaining sexual activity; agreeing to participate. Exclusion: having limitations on sexual activity by medical prescription.	Pregnant	Spain
Ferrari et al., (2013)	A qualitative study of women’s perceptions of provider advice about diet and physical activity during pregnancy	58	18–35	African-America; Caucasian; Latina	Inclusion: self-identified as Hispanic; Non-hispanic African American; Nonhispanic White; spoke English or Spanish; between 18 and 35 years old; between 27– 30 weeks of gestation.	Pregnant	USA
Findley et al., (2020)	Exploring women’s experiences and decision making about physical activity during pregnancy and following birth: a qualitative study	16	N/A	N/A	Inclusion: aged 18– 40 years old; lived in England; spoke fluent English; currently pregnant for the first time or had given birth to their first child less than three months before the recruitment date; were of a healthy weight (self-reported pre-pregnancy body mass index of 18.5 to 25).	Pregnant; within two years postpartum	England
Fletcher et al., (2022)	Isolation, marginalisation and disempowerment – understanding how interactions with health providers can influence smoking cessation in pregnancy	17	33, 24–43	Caucasian; Other	Inclusion: pregnant or had given birth to children in the northern suburbs of Adelaide (an area of low socioeconomic strata) in the previous 10 years; either smoked cigarettes or quit smoking cigarettes in pregnancy.	Pregnant; within two years postpartum; beyond the 1001 days	Australia
Fuss et al., (2022)	Attitudes and Communication Preferences for Vaccines among Pregnant Women Receiving Care at a Safety-net Hospital	28	25.3, 18–40	Black or African-American; Hispanic or Latinx; Caucasian; Hatian; Asian	Inclusion: English-speaking; pregnant women; aged 18 years and older; pregnancies of any gestational age; currently receiving prenatal care at the study site.	Pregnant	USA
Gamble et al., (2015)	Missed opportunities: A qualitative exploration of the experiences of smoking cessation interventions among socially disadvantaged pregnant women	6	18–38	N/A	Inclusion: smoking daily; being pregnant; experience of at least one health-worker-delivered intervention for smoking cessation during their pregnancy; residing in a metropolitan area of Adelaide within the lowest quintile of the Social-Economic Index for Areas index.	Pregnant	Australia
Garnweidner et al., (2013)	Experiences with nutrition-related information during antenatal care of pregnant women of different ethnic backgrounds residing in the area of Oslo, Norway	17	28	Norwegian; Algerian, Albanian, Pakistani, Thai, Turkish, Russian, Sri Lankan; Somalian.	Inclusion: age 16 years or older; first pregnancy; normal pregnancy; a pre-pregnancy Body Mass Index above 25 kg/m2.	Pregnant	Norway
Görig et al., (2015)	Screening for gestational diabetes mellitus in Germany: A qualitative study on pregnant women’s attitudes, experiences, and suggestions	20	32.6, 27–41	N/A	Inclusion: at least gestational week 29 (i.e., those who should have already been screened for GDM). Exclude: younger than 18 years old; without a telephone connection (landline or mobile); not fluent in German.	Pregnant	Germany
Grant et al., (2019)	Understanding health behaviour in pregnancy and infant feeding intentions in low-income women from the UK through qualitative visual methods and application to the COM-B (Capability, Opportunity, Motivation-Behaviour) model	10	29, 24–34	N/A	Inclusion: less than 30 weeks pregnant at the time of their first interview; pregnant; resident in areas of the highest quintile of deprivation according to the Welsh Index of Multiple Deprivation; claiming means tested (welfare) benefits.	Pregnant	Wales
Grenier et al., (2020)	Be Healthy in Pregnancy: Exploring factors that impact pregnant women’s nutrition and exercise behaviours	66	31.3	European; Mixed other; Unknown	N/A	Pregnant	Canada
Groves et al., (2010)	The complexity of consent: women’s experiences testing for HIV at an antenatal clinic in Durban, South Africa	25	27.46 (*n* = 13) 26.71 (*n* = 7) 29.80 (*n* = 5)	South African	Inclusion: pregnant within the last year.	Within two years postpartum	South Africa
Harrison et al., (2019)	Women with gestational diabetes mellitus want clear and practical messages from credible sources about physical activity during pregnancy: a qualitative study	27	32	Australian; Asian; Other	Inclusion: diagnosed with gestational diabetes mellitus; aged 18 to 40 years; singleton pregnancy; a normal 18-week ultrasound scan; able to express their thoughts in English. Exclusion: a high-risk pregnancy; conditions affecting their ability to participate in physical activity.	Pregnant	Australia
Haugland et al., (1996)	The pregnant smoker’s experience of ante-natal care — results from a qualitative study	33	20–35+	N/A	Inclusion: were daily smokers during the last three months before conception and still smoked regularly at ultrasound screening (still smoking in the 16th-18th week of pregnancy).	Pregnant	Norway
Helmersen et al., (2021)	Women’s experience with receiving advice on diet and Self-Monitoring of blood glucose for gestational diabetes mellitus: a qualitative study	12	24–41	Immigrant background; Norwegian	Inclusion: currently pregnant; diagnosed with gestational diabetes mellitus; experiences with gestational diabetes mellitus care in primary health care and secondary health care; Norwegian speaking.	Pregnant	Norway
Heslehurst et al., (2016)	Lived experiences of routine antenatal dietetic services among women with obesity: A qualitative phenomenological study	15	N/A	White	Inclusion: pregnant; a booking body mass index ≥ 30 kg/m2; attending an obesity-specific antenatal dietetic service.	Pregnant	UK
Hocking et al., (2020)	Women’s experiences of messages relating to alcohol consumption, received during their first antenatal care visit: An interpretative phenomenological analysis	12	22–41	N/A	Inclusion: attended an initial antenatal care visit within the previous two years.	Within two years postpartum	Australia
Jacobson et al., (2018)	Pioneer baby: suggestions for pre- and postnatal health promotion programs from rural English and Spanish-speaking pregnant and postpartum women	35	18–45	White; Hispanic; White Hispanic; Other	Inclusion: at least 18 years old; able to read and understand English or Spanish; able to give informed consent; at any stage of pregnancy or were within 6 weeks of their baby’s birth date.	Pregnant; within two years postpartum	USA
Jarlenski et al., (2016)	Pregnant Women’s Access to Information About Perinatal Marijuana Use: A Qualitative Study	26	19–36	African-Amercan; White; Hispanic/Latina; Other/Multiple races	Inclusion: either reported or tested positive for any substance used tobacco, alcohol, illicit drugs during an earlier part of the parent study.	Pregnant	USA
Jarvie (2017)	Lived experiences of women with co-existing BMI ≥ 30 and Gestational Diabetes Mellitus	27	30, 19–43	N/A	Inclusion: pregnant; having co-existing maternal obesity and gestational diabetes mellitus.	Pregnant	UK
Jessa & Hampshire (1999)	Use of folic acid by pregnant British Pakistani women: a qualitative pilot study	13	18–38	Pakistani	Inclusion: pregnant; be a Pakistani women; 18 years or older.	Pregnant	UK
Johnson et al., (2019)	Lived experiences of young pregnant women who smoke	5	18–20	White British	Inclusion: aged 18–20 years; pregnant or have given birth within the last year; smoked tobacco at some point during pregnancy.	Pregnant; within two years postpartum	UK
Knight-Agarwal et al., (2020)	The nutrition-related information seeking behaviours and attitudes of pregnant women with a high BMI: A qualitative study	28	N/A	N/A	Inclusion: a BMI of more than or equal to 30 kg/m2; aged 18 years or older; gestation of more than or equal to 12 weeks.	Pregnant	Australia
Knight-Agarwal et al., (2016)	The perspectives of obese women receiving antenatal care: A qualitative study of women’s experiences	16	N/A	N/A	Inclusion: a BMI of more or equal to 30 kg/m2; aged 18 years or older; of at least 12 weeks gestation; were accessing care through the local area health service.	Pregnancy	Australia
Lawrence et al., (2021)	New Zealand women’s experiences of managing gestational diabetes through diet: a qualitative study	18	34, 28–41	Tongan; Indian; Korean; Chinese; German; Brazilian; New Zealand European; Vietnamese; Māori; Cook Islands Māori; Filipino	Inclusion: currently pregnant; a diagnosis of GDM made before 30 weeks’ gestation. Excluded: pre-existing diabetes mellitus; under the age of 16 years; unable to adequately understand verbal explanations in English; had special communication needs.	Pregnant	New Zealand
Legault et al., (2014)	Nutrition Information-seeking Behaviour of Low-income Pregnant Maghrebian Women	14	32	Moroccan; Algerian	Inclusion: being a French-speaking immigrant from the Maghreb; living in Montreal; primigravid; at least 18 years old; with a household income below before tax low income cut offs. Exclusion: a multiple pregnancy.	Pregnant	Canada
Luo et al., (2024)	The influence of Chinese culture and customs on the beliefs and health-related behaviours of Chinese women with gestational diabetes mellitus: A qualitative study	15	31, 25–38	N/A	Inclusion: aged ≥ 18 years; diagnosed with GDM; no other pregnancy complications; able to speak fluent native Mandarin without speech disorders.	Pregnant	China
Lynch et al., (2012)	Pregnant and Recently Pregnant Women’s Perceptions about Influenza A Pandemic (H1N1) 2009: Implications for Public Health and Provider Communication	144	18–44	White; Black; Asian American; Multiracial; American Indian or Alaskan Native	Inclusion: pregnant or recently pregnant (within 6 months postpartum); 18 years or older.	Pregnant; within two years postpartum	USA
Malik et al., (2021)	Pregnant women’s perspectives about maternal immunization in Latin America	162	27, 18–42	Argentinian; Brazilian; Honduran; Mexican; Peruvian	Inclusion: 18 years of age or older; pregnant at the time of the focus group; had at least one prenatal visit.	Pregnant	Argentina; Brazil; Honduras; Mexico; Peru
Martinelli et al., (2021)	Alcohol Consumption During Pregnancy in Brazil: Elements of an Interpretive Approach	14	20->35	Black; White; Mixed race	N/A	Pregnant; within two years postpartum; beyond the 1001 days	Brazil
McKenzie et al., (2022)	Risk Perceptions about Cannabis Use and Receipt of Health-Related Information during Pregnancy	21	34.4	African American; White; Multiracial	Inclusion: age 16–50 years; ability to communicate in English; intent to deliver at Ohio State University Wexner Medical Centre.	Pregnant	USA
McTigue et al., (2022)	Contraceptive trajectories postpartum: A longitudinal qualitative study of women living with HIV in Cape Town, South Africa	30	29, 19–45	Black African	Inclusion: 18 years or older; 32–35 weeks pregnant; living with HIV; currently prescribed antiretroviral therapy; English or isiXhosa speaking. Exclusion: those whose pregnancies were considered high risk for reasons other than human immunodeficiency virus status (e.g., preeclampsia, preterm labour); those involved in other antiretroviral therapy adherence-related studies; those unable to provide informed consent.	Pregnant; within two years postpartum;	South Africa
Minjing et el., (2021)	Qualitative Study on the Health Education Needs of Pregnant Women with Gestational Diabetes	12	32.3, 25–38	N/A	Inclusion: diagnosed with gestational diabetes based on the diagnostic criteria recommended by the International Association of Diabetes and Pregnancy Study Group; normal cognitive communication ability; voluntary participation with informed consent. Exclusion: diagnosed with diabetes before pregnancy; history of stillbirth or fetal death; multiple pregnancies; diagnosed with fetal abnormalities.	Within two years postpartum	China
Nagourney et al., (2019)	Obese women’s perceptions of weight gain during pregnancy: a theory-based analysis	21	18–33	African American	Inclusion: in the first or second trimester of pregnancy; aged 18 years or older; had not yet received nutritional counselling.	Pregnant	USA
Nguyen-Hoang et al., (2024)	Barriers and enablers toward healthy eating and weight gain among pregnant women in Vietnam: A qualitative study with analysis informed by the theoretical domains framework and COM-B model	20	28	N/A	Inclusion: pregnant women aged 18 or older; residing in Vietnam; without any diagnosed medical condition requiring dietary or medication recommendations.	Pregnant	Vietnam
Nielsen et al., (2015)	Reasons for women’s non-participation in follow-up screening after gestational diabetes	7	27–36	Caucasian; Asian	Inclusion: been registered with gestational diabetes between 1 June 2012 and 1 June 2013; had given birth between one and two years previous to those dates.	Within two years postpartum	Denmark
Ningrum et al., (2024)	Experiences of Low-Income Indonesian Pregnant Women Regarding the Challenges of Receiving Health Services: A Qualitative Content Analysis	17	22–39	N/A	Inclusion: pregnant women in their 3rd trimester, postpartum women within 1–42 days after labor, or mothers of babies aged 0–1 year; confirmation of their pregnancy through village midwives’ services; adherence to pregnancy check-ups from the village midwives.	Pregnant; within two years postpartum; beyond the 1001 days	Indonesia
Okafor et al., (2020)	Applying the Ecological Model to understand pregnant women’s perspectives on the modifiable constraints to physical activity during pregnancy A qualitative research study	15	29.4	Black; Coloured	Inclusion: pregnant women; attending antenatal care at the study sites (Primary health clinics); single pregnancy; all level of trimester. Exclusion: <18 years of age; pregnancy complications (hypertension); persistent excessive shortness of breath; severe chest pain; regular and painful uterine contractions; vaginal bleeding; disabilities or pre-existing health conditions; preventing the effect of physical activity; unable to speak English.	Pregnant	South Africa
Oxlad et al., (2023)	The Complexities of Managing Gestational Diabetes in Women of Culturally and Linguistically Diverse Backgrounds: A Qualitative Study of Women’s Experiences	33	20–42	Indian; Australian; Caucasian; English; Pakistani; Aboriginal; Bangladeshi; Colombian; Filipino/Italian; Filipino/Spanish; Iranian; Iraqi; Nepalese	Inclusion: a GDM diagnosis within the past 12 months; the ability to speak and understand English.	Within two years postpartum	Australia
Padmanabhan et al., (2015)	A qualitative study exploring pregnant women’s weight-related attitudes and beliefs in UK: the BLOOM study	19	19–38	White; Other	Inclusion: still participating in the parental longitudinal study; were in their 3rd trimester.	Pregnant	England
Palombarini et al., (2014)	Nutritional practices of expectant mothers supported by a Family Health Unit: an exploratory study	12	18–27	N/A	Inclusion: in the second trimester or later of their first pregnancy; 18 years old or older; no record of previous disease (diabetes, hypertension, heart diseases) or any other adverse condition that would require a specialist dietary routine; registered and supported by a family health unit in a medium-size city.	Pregnant	Brazil
Pardell-Dominguez et al., (2021)	The meaning of postpartum sexual health for women living in Spain: a phenomenological inquiry	10	33	Catalonian	Inclusion: to have given birth for the first time within the last year in a Catalonian hospital. Exclusion: < 18 years old; gave birth to more than one infant (twins or triplets); did not speak Catalan or Spanish languages; had cognitive disabilities.	Within two years postpartum	Spain
Patterson et al., (2023)	Using the COMB framework to elucidate facilitators and barriers to COVID19 vaccine uptake in pregnant women: a qualitative study	13	18–45	N/A	Inclusion: new/expectant mothers aged 18—45 years who had either: (a) had a pregnancy since April 2021 or (b) were known to be pregnant at the time of recruitment.	Pregnant	Northen Ireland
Pearlman Shapiro et al., (2022)	Breastfeeding and contraception counseling: a qualitative study	20	25.8, 19–41	Latinx; Black; White; Asian	Inclusion: were 14 years of age or older; were on the Albert Einstein Weiler Hospital postpartum service; spoke English.	Within two years postpartum	USA
Pledger (2015)	Exploring the experiences of pregnant women using an NHS stop smoking service: a qualitative study	6	N/A	N/A	Inclusion: referred to the NHS stop smoking service whilst pregnant (July 2012-13); required to speak English fluently; be over the age of 18; had an initial assessment with an NHS stop smoking advisor. Exclusion: were referred to the NHS stop smoking service but did not meet with an NHS stop smoking advisor; do not speak fluent English and would require an interpreter; accessed the service whilst pregnant before July 2012; have accessed mental health services within the preceding 12 months.	Pregnant	UK
Potgieter et al., (2018)	Factors influencing post-partum women’s choice of an implantable contraceptive device in a rural district hospital in South Africa	10	23–35	N/A	Inclusion: postpartum women who delivered at Knysna Provincial hospital labour ward between February 2015 and August 2015. Exclusion: were unable or did not consent; did not speak English, Afrikaans or Xhosa; were younger than 18 years of age (due to consent), or had any contraindications to the implantable contraceptive device.	Within two years postpartum	South Africa
Rahmawati et al., (2021)	Nutrition information-seeking behaviour of Indonesian pregnant women	23	18–37	Indonesian	Inclusion: ≥ 12 weeks of gestation; age ≥ 18 years; singleton pregnancy, living in Malang City for a minimum of two years; apparently healthy and a Bahasa speaker.	Pregnant	Indonesia
Raymond et al., (2009)	Pregnant women’s attitudes towards alcohol consumption	20	33, 23–40	N/A	Inclusion: currently pregnant women engaged in antenatal care.	Pregnant	UK
Sloan et al., (2016)	Pregnant Women’s Experiences and Views on an “Opt-Out” Referral Pathway to Specialist Smoking Cessation Support: A Qualitative Evaluation	18	24, 18–33	White British; White European	Inclusion: attending an ultrasound “dating” scan appointment from August to November 2013; CO levels at least 4 parts per million.	Pregnant; within two years postpartum	UK
Snyder et al., (2020)	A mixed-methods investigation of women’s experiences seeking pregnancy-related online nutrition information	10	23–35	European/Canadian/White	Inclusion: 18 years of age or older; resided in Ontario, Canada; able to read and speak English; be pregnant at the time of completion (for survey) and be pregnant or up to 3 months post-partum (for interview); be primiparous.	Pregnant; within two years postpartum	Canada
Söderbäck et al., (2023)	Barriers to using postpartum family planning among women in Zanzibar, Tanzania	24	20–44	N/A	Inclusion: a pregnancy and childbirth without complications; age above 18 years; a woman in overall good health.	Within two years postpartum	Tanzania
Stacey et al., (2022)	‘I don’t need you to criticise me, I need you to support me’. A qualitative study of women’s experiences of and attitudes to smoking cessation during pregnancy	19	21–40	White British	Inclusion: had smoked whilst pregnant in the last five years and who lived in the local area.	Within two years postpartum; beyond the 1001 days	UK
Stengel et al., (2012)	“What My Doctor Didn’t Tell Me”: Examining Health Care Provider Advice to Overweight and Obese Pregnant Women on Gestational Weight Gain and Physical Activity	24	21–35	White; Other	Inclusion: active participants of the Penn State First Baby Study; had given birth to their first child; aged between 18 and 35 years; being overweight (body mass index [BMI] 25.0–29.9 kg/m2 ) or obese (BMI more than or equal to 30.0 kg/m2 ) before pregnancy; had a singleton pregnancy; English speaking. Exclusion: gestational weight gain was less than 5 pounds.	Within two years postpartum	USA
Sundstrom et al., (2018)	“My Body. My Choice”: A Qualitative Study of the Influence of Trust and Locus of Control on Postpartum Contraceptive Choice	47	18–39	N/A	Inclusion: 18 years old or older; receiving postpartum care at an outpatient clinic; English speaking	Within two years postpartum	USA
Svensson et al., (2018)	What is the postpartum experience of Danish women following gestational diabetes? A qualitative exploration	5	20–38	N/A	Inclusion: women 3–5 months after delivery diagnosed with gestational diabetes mellitus.	Within two years postpartum	Denmark
Tod (2003)	Barriers to smoking cessation in pregnancy: a qualitative study	11	19–38	N/A	Inclusion: over 16 years of age; able to give informed consent; were able to participate during the study period; had smoked during their pregnancy.	Pregnant	UK
Walker et al., (2020)	It’s not easy” — A qualitative study of lifestyle change during pregnancy	17	30.9	Australian; Indian; New Zealander; Mauritian; Saudi Arabian; Scottish; Singaporean; Sri Lankan	Inclusion: pregnant; relatively low-risk pregnancy, receiving routine maternity care consisting of approximately 10 outpatient visits led by midwives and with obstetric oversight.	Pregnant	Australia
Wan et al., (2019)	Ethnic Differences in Dietary Management of Gestational Diabetes Mellitus: A Mixed Methods Study Comparing Ethnic Chinese Immigrants and Australian Women	83	31.9 (Chinses) 33.3 (White)	Chinese; White	Inclusion: self-identified Chinese and white women with gestational diabetes mellitus.	Pregnant	Australia
Watson et al., (2016)	“Just because you’re pregnant, doesn’t mean you’re sick!” A qualitative study of beliefs regarding physical activity in black South African women	13	28, 19–41	Black African	Inclusion: black pregnant women; non-complicated pregnancies; in the third trimester.	Pregnant	South Africa
Wennberg et al., (2013)	Women’s experiences of dietary advice and dietary changes during pregnancy	23	19–41	N/A	Inclusion: expecting their first child; currently mid-pregnancy.	Pregnant	Sweden
Woodruff et al., (2021)	Pregnant people’s experiences discussing their cannabis use with prenatal care providers in a state with legalized cannabis	33	29	Black; Latinx; White; More than one race; American-Indian; Asian/Pasic Islander; Declined to state	Inclusion: currently pregnant or had been pregnant within the last year; had used cannabis regularly (at least monthly) in the last year or in the year before their most recent pregnancy; were 18 years or older; lived in California; and were English-speaking.	Pregnant; within two years postpartum	USA
Yuen et al., (2016)	Perceptions of Hong Kong Chinese women toward influenza vaccination during pregnancy	32	23–35+	Chinese	Inclusion: 18 years of age or older; Cantonese speaking; Hong Kong residents; recent birth of a live newborn.	Within two years postpartum	China

Note. N/A = Not applicable due to lack of information reported. *Age is presented as the study has reported; therefore, age can be presented as an average (represented by a single number) or as a range (represented by two numbers separated by a dash). All studies are confirmed to have participants aged 18 years or older. ** Where in the 1001 days perinatal care timeframe are the participants at the time of data collection, either currently pregnant; currently within two years postpartum; or currently beyond the 1001 days

There was a range of quality across the studies, with 44 being categorised as high, 16 as medium, and 21 as low. A complete summary of the CASP qualitative assessment of all included studies and their final quality category are included in the supplementary materials.

From the data extracted from these 81 records, three themes were developed to identify women’s experiences of discussing health behaviours within their maternity care interactions. Theme 1 *‘Women believe they lack knowledge of health behaviour’*, theme 2 *‘Women want a discussion about health’* and theme 3, *‘A successful health discussion can positively impact the woman-HCP relationship’*. Table 5 shows which records contributed to each theme.

**Table 5:** A matrix table to show how each record contributed to the final findings

Record	Findings								
	Theme 1 Women believe they lack knowledge of health behaviour.			Theme 2 Women want a discussion about health.			Theme 3 HCP communication style can impact how engaged women are in a health discussion.		
	Influences on the feeling of being knowledgeable.	Feeling unknowledgeable about health can lead to distress.	Health information can feel inaccessible.	Timing of health behaviour discussion is important.	Dialogue between women and HCPs is valuable.	It is effortful for women to initiate and maintain a health discussion.	Passive communication style can discourage engagement in health conversation.	Listening to and then responding to women’s needs can encourage engagement in health conversations.	Accessible conversations about health can rebuild trust between a woman and HCP.
Arreciado Maranon et al. (2022)	X								X
Atkinson et al. (2016)		X							
Atkinson and McNamara (2017)		X							
Barbosa-Leiker et al. (2020)				X					
Baron et al. (2017)	X				X	X	X	X	
Beckham et al., (2015)							X		
Bianchi et al. (2016)		X				X			
Bianchini et al., (2020)		X				X			
Bookari et al. (2017)			X						
Booth et al., (2018)			X	X					
Boydell et al., (2020)		X							
Bradford et al., (2024)			X		X				X
Bulman et al., (2013)				X					
Chana & Haith-Cooper (2019)		X			X		X		X
Congdon et al., (2020)				X					
Cronin et al., (2023)						X		X	
Cunningham et al., (2018)					X				
Dinsdale et al., (2016)			X			X			
Draffin et al., (2016)		X							
English & Greyson (2022)				X					
Fernández-Sola et al., (2018)				X					
Ferrari et al., (2013)			X						
Findley et al., (2020)							X		
Fletcher et al., (2022)							X		X
Fuss et al., (2022)					X				
Gamble et al., (2015)		X			X		X		
Garnweidner et al., (2013)	X					X			
Görig et al., (2015)						X			
Grant et al., (2019)		X							
Grenier et al., (2020)				X					
Groves et al., (2010)									X
Harrison et al., (2019)		X							
Haugland et al., (1996)	X								
Helmersen et al., (2021)								X	
Heslehurst et al., (2016)					X			X	
Hocking et al., (2020)	X								
Jacobson et al., (2018)								X	
Jarlenski et al., (2016)			X						
Jarvie (2017)							X		
Jessa & Hampshire (1999)					X				
Johnson et al., (2019)					X				
Knight-Agarwal et al., (2020)		X							
Knight-Agarwal et al., (2016)					X				
Lawrence et al., (2021)			X		X				X
Legault & Marquis (2014)									X
Luo et al., (2024)			X						
Lynch et al., (2012)		X							
Malik et al., (2021)		X	X						
Martinelli et al., (2021)						X			
McKenzie et al., (2022)			X				X		
McTigue et al., (2022)						X			
Minjing et el., (2021)		X			X				
Nagourney et al., (2019)					X		X	X	
Nguyen-Hoang et al., (2024)	X								
Nielsen et al., (2015)		X			X		X		
Ningrum et al., (2024)				X		X			
Okafor et al., (2020)	X								
Oxlad et al., (2023)			X						
Padmanabhan et al., (2015)			X						
Palombarini et al., (2014)					X				
Pardell-Dominguez et al., (2021)				X					
Patterson et al., (2023)		X	X				X		
Pearlman Shapiro et al., (2022)		X							
Pledger (2015)		X							
Potgieter et al., (2018)			X				X		
Rahmawati et al., (2021)		X							X
Raymond et al., (2009)		X				X			
Sloan et al., (2016)							X		
Snyder et al., (2020)	X						X		
Söderbäck et al., (2023)	X								
Stacey et al., (2022))								X	
Stengel et al., (2012)	X					X			
Sundstrom et al., (2018)						X		X	X
Svensson et al., (2018)		X							
Tod (2003)			X				X		
Walker et al., (2020)	X			X					
Wan et al., (2019)	X				X				
Watson et al., (2016)		X			X				
Wennberg et al., (2013)						X			
Woodruff et al., (2021)								X	
Yuen et al., (2016)					X				

### Theme 1: Women believe they lack knowledge of health behaviour

Women who are motivated to make health behaviour change sometimes feel that they lack the information resources to achieve this. This is exacerbated by the confusing and inconsistent information delivery style and inaccessibility of the health information itself. This belief can cause distress for women and weaken their trust in HCP as a source of health information. This experience takes place over three subthemes.

#### Subtheme: Influences on the feeling of being knowledgeable

There are individual differences between women’s internal motivation and intention to make health behaviour changes during the maternity period [38, 39]. For example, women who did not intend to quit smoking during pregnancy stated that they were satisfied with the health information regarding smoking, whereas in the same study, women who expressed a desire to stop smoking were not sufficiently satisfied with the same information in [40].

The unique differences for each woman’s life circumstance and background can also impact how women interpret the same health information. When health information conflicted with an established cultural norm, that information was perceived as difficult to understand and implement [41]. In contrast to this, information that was easy to implement (and therefore more coherent within one’s cultural norms) was perceived to increase one’s knowledge and self-confidence [42]. Clearly, the influence of one’s internal state of motivation, and its connectedness to the environment externally, can have an impactful influence on feelings of capability and knowledgeability to attempt a health behaviour change.

*According to my experience*,* what they (health professionals) discussed are all about the dietary habit of women here (in Australia)*,* so if the suggestions could be more relevant to Chinese lifestyle*,* a lot of things that have been taught currently are not meaningful. Participant quote from Wan*,* Teede* [41].

#### Subtheme: Feeling unknowledgeable about health can lead to distress

Women often felt that there could be more health behaviour information relevant to their care that was not being sufficiently communicated to them by HCPs [43–46]. For behaviours such as alcohol consumption and vaccine taking, ambiguous information was sometimes interpreted by women as HCPs not knowing how much risk these behaviours actually posed [47, 48].

*I did ask my midwife and my health visitor at the time [about potential risks post insertion of contraception] and nobody was that clear on the process […] it didn’t seem that people were particularly certain about how things worked. Participant quote from Boydell*,* Cooper* [49].

Due to maternity care including interactions with various different HCPs and little continuity of care, women found that information delivery across different HCPs could be conflicting and inconsistent [50, 51]. Some women believed that health information was purposefully not delivered to them because HCPs assumed that women already possessed certain knowledge about diet, exercise and smoking, either because it was considered common knowledge [52] or because they had been through pregnancy before [53]. Therefore, this perceived lack of health knowledge caused distress to many women as they expressed a fear of not having sufficient knowledge resources to make healthy decisions [54, 55].

*Among doctors*,* there are two sorts of stories: some say yes*,* others no. […] my gynaecologist is against dietary supplements*,* but when I arrived at the maternity hospital*,* [the midwife] gave me a prescription [to take dietary supplements]. What am I expected to do? Participant quote from Bianchi*,* Huneau* [56].

#### Subtheme: Health information can feel inaccessible

Even when women confirmed they received a wide variety of health information they still expressed fear of lacking knowledge about health [57–59]. Women believed that they did not have access to all the health information delivered to them by HCPs because an overwhelming amount of the health information felt difficult to retain often felt too vague to be practically applicable [60, 61].

*None of my doctors*,* like my obstetrician or my GP*,* neither of them really have said specifically what to do. They just sort of said*,* “Oh you know eat a healthy diet”*,* but they haven’t said what [that is]. Participant quote from Bookari*,* Yeatman and Williamson* [62].

For some women, these combined experiences of confusing information delivery and distress surrounding inaccessible health information culminated in them expressing distrust towards the HCP [63, 64]. The HCP was no longer considered a credible source of information and so any health information provided was then perceived as irrelevant [65]. This in turn led some women to seek their own health information outside of their care interactions [43] or give greater weight to their own personal experience compared with opposing HCP-delivered advice [66].

*Nian Zhen reported being given a pamphlet by the dietitian that she “can’t follow” because of high blood glucose readings after consuming the foods listed. She went on to say the glucometer was the only thing she could trust. Participant quotes from Lawrence*,* Ward* [64].

### Theme 2: Women want a discussion about health

To address their perceived lack of knowledge about health, women expressed a want for collaborative discussions about health behaviour change with their HCP. The timing of health behaviour change discussions within existing care interactions and the level of engagement from the HCP during this discussion can be influential on the woman’s perceived success of the discussion. This is explored in three subthemes.

#### Subtheme: Timing of health behaviour discussion is important

A women’s individual intention to change behaviour has an impact on when and where they would prefer to have health behaviour discussions [67]. Whilst some health information delivery is almost unanimously agreed upon (for example, discussions about restarting contraceptives are preferred in postnatal care) [68]; other advice was wanted only when it was deemed relevant and useful – a timeframe which cannot be easily defined or generalised across all women [69]. Listening to when women are open to receiving advice seems to be the way in which women want to be approached for health behaviour discussion [57].

*I just got [nutrition counselling] from my doctor last week*,* so they gave you the information but it’s kind of late*,* like I’m already six months now*,* so it was just a little late to be getting that information. Participant quote from Grenier*,* Atkinson* [69].

Having a conversation about health as part of existing maternity care appointments was viewed as the most accessible way that women wanted HCPs to initiate this discussion [67]. However, some women expressed feelings of embarrassment and even fear when engaging in these discussions, suggesting these are delicate conversations that need to be conducted with sensitivity [70, 71].

*Participants reported that they often did not disclose cannabis use to healthcare providers because of stigma surrounding the issue and feared legal repercussions. Participant experience from Barbosa-Leiker*,* Burduli* [72].

#### Subtheme: Dialogue between women and HCPs is valuable

Women wanted more information whilst they are in a state of openness to receiving advice [55, 73]. Maternity care is an opportunity for women to reflect on their health behaviour outside of and beyond their pregnancy, and the desire for information beyond the routine is a reflection of this motivation to make lasting changes to their health [44, 64].

*[Women] saw pregnancy as a time to start making positive changes to their lifestyle. One participant stated: […] me and my partner straight away thought we have to eat a lot better. Participant quote from Knight-Agarwal*,* Williams* [74].

There is a preference for a face-to-face discussion over receiving written information because there is the opportunity for a dialogue [42, 51, 75]. Women explained that they want to ask questions and learn the reasons that behaviour change are beneficial for their own and their baby’s health [76]. Having had a health discussion can improve a woman’s confidence in their capability to make healthy choices which in turn relieves feelings of distress [41, 46]. In some cases, women felt that HCPs over relied on written resources to provide this desired health information [77].

*I noticed that there were promotion flyers available at the maternal and child health center. Even if I had read it. my confidence would not suddenly be increased. I would still require an explanation from the professionals […] If you receive it [the vaccine] just because you have read a piece of paper*,* it seems like I am treating it as trivial. So*,* if someone has explained it to me and balanced the risks for me*,* I will have more trust in it. Participant quote from Yuen*,* Dodgson and Tarrant* [77].

#### Subtheme: It is effortful for women to initiate and maintain a health discussion

Across maternity care interactions, women felt there was a lack of disclosure opportunities in which they could bring up the topic of health to their HCP, and when they were able to disclose to their HCP the opportunity for discussion went untaken [48, 78]. Despite this, women continued to ask their HCP questions but with the caveat that now they only felt able to ask questions if there was a problem that needed fixing [56]. This is troubling, as the act of asking questions is something that many women highlighted as how they understood important health information that otherwise felt inaccessible to them [58, 79]. When women are successful at initiating a health discussion, they expressed that the conversations were often brief due to time constrains and that the discussion was rarely followed up in subsequent interactions [38, 80].

*A few women mentioned they had called their midwives*,* especially when they were worried about having eaten or done something harmful […] Women were sometimes hesitant*,* however*,* to call their midwives about questions related to health behaviours*,* because they didn’t want to bother the midwife unnecessarily. Participant experience from Baron*,* Heesterbeek* [42].

### Theme 3: HCP communication style can impact how engaged women are in a health discussion

How the HCP communicates health information to the woman during a discussion can be an important factor in the perceived success of the conversation. If the HCP is perceived to be communicating health information passively and not making the woman feel like a priority during their interactions, then this can lead to feelings of frustration and a breakdown in the process of shared decision making. Comparatively, when women feel actively engaged in the discussion and health information is adapted to their level of accessibility then a trusting patient-provider relationship can form which has a positive impact on the process of shared decision making. This experience is captured in three subthemes.

#### Subtheme: Passive communication style can discourage engagement in health conversation

How a HCP chooses to communicate health information can sometimes discourage women from engaging in the process of shared decision making [59]. When information was communicated to women passively, interactions felt generic or insincere [81], with women describing these experiences almost like a script or a tick box exercise [63, 82]. This resulted in health conversations where women did not feel like their health was a priority to their HCP, either because baby’s health was prioritised instead [83] or women’s health information was not brought up for discussion [51]. This left women feeling unclear how much support they should expect to receive from their HCP about making a health behaviour change [42]. Women became frustrated with HCPs who took this approach to the health discussion [46]. This resulted in women feeling judged for their health choices and even pressured by HCPs to make certain health choices [55, 66].

*[The healthcare provider] never asked me if I wanted to [be referred]*,* she just told me that she was referring me […] She made me feel like I had no choice […] like I didn’t have a voice […] but then*,* you know I should have a choice whether or not I want to go. There was no discussion. Participant quote from Sloan*,* Campbell* [84].

#### Subtheme: Listening to and then responding to women’s needs can encourage engagement in health conversations

When HCPs made an effort to actively engage women in discussion about their health, women felt more positively about the outcome of the conversation [85]. HCPs who advocated for women to make their own decisions about their health were appreciated, and this helped to foster a willingness from women to be more open and honest during the discussion [79]. Specifically, when HCPs took the time to ask women questions about their current health and adapted health information to their existing lifestyle, women expressed feeling more respected and more supported to make a successful health behaviour change [42, 79].

*[A healthcare provider] would stay for like two hours and just talk about my health and what I am doing and how I am feeling*,* and I just felt like she was really focused on me. Participant quote from Jacobson*,* Zackula* [86].

HCPs also tailored health information to a woman’s individual level of knowledge accessibility [79]. For example, making advice clear and direct and even presenting information in the woman’s preferred language [48, 87]. This continuing effort to make women feel respected and supported had an influence on a woman’s desire to build a relationship with their HCP [75].

*I felt more comfortable with [the nurses]. Like they would tell us*,* we’re not telling you to stop completely*,* but maybe decrease your intake. Like if you smoke five blunts a day*,* maybe try smoking four*,* and then just trying to gradually decrease. Participant quote from Woodruff*,* Scott and Roberts* [88].

#### Subtheme: Accessible conversations about health can rebuild trust between a woman and HCP

Having an accessible health discussion with a HCP can rebuild feelings of trust [64]. A HCP can regain their position as a trusted source of information for women through respectful and practically supportive conversation [79]. Women’s feeling of trust increased as HCPs continued to actively listen and work with a woman’s individual needs [82]. Continuity of care with a trusted HCP was considered valuable, and trust had an impact on women’s intention to make changes to their health behaviour [51, 79, 89].

*I like her a lot. I trust her a lot*,* yes. She has given me a lot of confidence and I’ve seen that she’s*,* I don’t know… She cares a lot about her patients. So*,* I let her guide me*,* and if she tells me to do something*,* I believe that it’s good for me and my baby. Participant quote from Arreciado Maranon*,* Fernández-Cano* [90].

## Conclusions

The current review brings together the broad literature of women’s experiences of discussing health behaviours within their maternity care interactions. Women believe they lack knowledge of health behaviour; due to the inconsistent information delivery style and inaccessibility of the health information itself all of which contributed to feelings of distress. To resolve these feelings, women expressed a want for collaborative discussions about health behaviour change with their HCP, with an emphasis on wanting time to ask questions. During these discussions, how the HCP communicates can impact how engaged women are in the conversation. Passive delivery of information led to feelings of frustration for women, whereas, when information was communicated accessibly and women were involved in their health decision making, then trust could be built between the woman and her HCP. The dual impact of accessible health conversation and a trusting rapport with the HCP helped women to feel more confident in their knowledge of health behaviour and consequently their ability to make healthy choices.

The current review provides support for Smith and Lavender [8], Smith, Taylor and Lavender [18] in that it is apparent that women are open to making behaviour changes, especially in the postnatal period, but are limited at times by a lack of confidence in their health knowledge and in part a lack of accessible health information. The current review has made progress in identifying what support women would like from HCPs to resolve these issues. Talbot, Strong [20] highlights the potential for roles like the GP to act as the professionals providing accessible information, despite some GPs feeling that their role was primarily to refer women to other resources and not to provide a dialogue. We now know women want a dialogue because the opportunity to ask questions is key in their process of understanding health information. However, since Smith, Taylor and Lavender [18] found that women felt like they had successfully achieved behaviour change as a result of learning more about health it suggests that policy needs to more clearly highlight opportunities and resources for HCPs, like GPs, to capitalise on the TMs that they and the women recognise. Women want a dialogue with a HCP they trust to communicate in an accessible way and with knowledge that they trust. This aligns with existing global policy that promotes competency and confidence in delivering health messages throughout the pregnancy journey [7, 19]. If women are being open in communicating to their HCP when they have intentions to change health behaviour, and they would like HCP support with this through shared decision making and an accessible dialogue around health information, then this TM should be utilised. However, the lack of focus on pregnancy outcomes as a result of HCP-women conversations makes it challenging to suggest whether capitalising on these TMs is effective in supporting women to make a successful health behaviour change after the interaction. Further research should focus on investigating whether women make successful health behaviour changes after a HCP-woman interaction and how this interaction contributed to that change in behaviour. Furthermore, the current review supports Rockliffe, Peters [91] conclusion’s that non-psychological factors, such as accessibility of health information, have a meaningful impact on a women’s experience of health behaviour change and in doing so support the need for the COM-B and TM models to be used in tandem when exploring how best to support when to make effective health behaviour change within the pregnancy journey.

### Recommendation

Evidently, these health conversations are delicate with women’s own internal motivation to change and feelings of trust in the HCP as a source of information differing, suggesting there is not a one size fits all approach. External factors such as stigma around certain health behaviours, like alcohol consumption, smoking, and recreational drug use, can mean these conversations have the potential to be very emotive for women. How a HCP choses to communicate information about risk relating to these health behaviours can have a large impact on how the women feels once the interaction has ended, with the potential for real harm to be done to women’s motivation to change and their trust in a HCP if communication is not handled with sufficient sensitivity [70–72], Future research should use policy supported theory such as the Theoretical Domains Framework [92, 93] to understand more about healthcare provider behaviours and how to make successful behaviour change with interventions and training. Potential training could focus on the behaviour change techniques [94] instructing HCPs on how to conduct these conversations and building confident through self-talk as these areas have been identified as current challenges to successful conversations [20, 95, 96]. This need to upskill healthcare providers is especially relevant in maternity care right now in countries like the UK [97].

The findings from the current review highlight how health behaviour discussions can act as a TM [11, 14] for women and why women feel open to health messaging in these conversations within the 1001 days pregnancy journey. Women expressed that they feel they need trusted information to make an accurate risk perception before attempting to change their behaviour. Building feelings of distress and distrust in HCP due to inconsistent and confusing health information delivery could act as a barrier to successful behaviour change in this way. An example of this finding is when women felt that their own personal experience was more reliable than that of the HCP. This personal experience, because it is perceived as more reliable, was weighted as more impactful in the eyes of women when considering risks surrounding behaviour and so consequently unhealthy behaviours continued [72, 79, 98]. Having an open and respectful dialogue could in part resolve this issue. By having a respectful dialogue, women may be more likely to trust and accept the information provided to them [99]. Moreover, if the women initially disagree with any of the advice provided, they have the immediate opportunity to talk through their thoughts and feelings with a professional who is able to answer their questions [64]. This highlights how an open and respectful dialogue can act as a TM with the potential to inspire behaviour change in women during their perinatal interactions.

### Strengths and limitations

A strength of this review was the inclusion of reflexivity and stakeholder involvement, both of which were considered continuously throughout the research process in order to best serve the research question and identify women’s experiences as they are [100]. Empowering and giving a voice to stakeholder contributors has been shown to improve the quality and relevance of health research, in addition to aligning with the principles of citizenship, accountability, and transparency required by various research councils [101]. The impact this had on the current review was that health discussions across a range of behaviours was explored and in doing so certain experiences were more easily identified because of their prevalence across behaviours as opposed to if this review had placed limitations on the number or type of behaviours included. In this way, stakeholder insight was incredibly valuable to the creation of the current study’s search strategy. During the thematic synthesis the feedback provided by the stakeholders reinforced that the experiences of women were clear within the themes and reflected accurately. For example, the role of autonomy and respect within communication delivery was highlighted as important and reaffirmed that fear is too often used to make women make a ‘choice’. This feedback was useful and appreciated as during reflective practise researcher LH, who developed the themes, continuously made reflections on the impact of their background and specifically the fact that they have not worked as a healthcare clinician. Because of this background, researcher LH was aware of being potentially dismissive of the work healthcare clinicians do. Due to the stakeholder discussion, researcher LH felt able to include and highlight the experience of women having their autonomy disrespected as healthcare provider stakeholders were able to provide their own feedback and give their acceptance of the way in which the theme was expressed. Resultingly, the final themes have been reflexively considered and member checked to ensure experiences of women, and the actions of healthcare providers in the eyes of women, has been reported in a way which is respectful but accurate to the findings of the review.

In conclusion, within maternity care interactions, women initially experience health behaviour discussions through the lens of lacking knowledge of health behaviour, and to resolve this perceived lack of knowledge they want a discussion about health. During these discussions, the HCP communication style can impact how engaged women are in a health discussion, with women having a preference for accessible information and being involved in shared decision making. This suggests that healthcare providers should be engaging women in conversations where women can feel confident to express motivation for change honestly to the HCP, and then together they can both contribute to shared discussions about the woman’s health. Future research should focus on providing support for healthcare providers to achieve this through training and intervention.

## Supplementary Information

Supplementary Material 1.

Supplementary Material 2.

Supplementary Material 3.

Supplementary Material 4.

## Data Availability

All data generated or analysed during this study are included in this published article [and its supplementary information files].

## Abbreviations

HCP: Healthcare provider
TM: Teachable moment
COM-B: Capability Opportunity Motivation Behaviour
PRISMA: Preferred Reporting Items for Systematic Reviews and Meta-Analyses
GP: General Practitioner
CASP: Critical Appraisal Skills Programme

## References

[1] Department of Health and Social Care. The best start for life: a vision for the 1,001 critical days. In: Care DoHaS, editor. Gov.uk2021.

[2] World Health Organization. Guidelines for identification and management of substance use and substance use disorders in pregnancy. 2014. 24783312

[3] Conner M, Norman P. Health behaviour: Current issues and challenges. Psychol Health. 2017;32(8):895–906. 28612656 10.1080/08870446.2017.1336240

[4] Scarborough P, Bhatnagar P, Wickramasinghe KK, Allender S, Foster C, Rayner M. The economic burden of ill health due to diet, physical inactivity, smoking, alcohol and obesity in the UK: An update to 2006–07 NHS costs. J Public Health. 2011;33(4):527–35. 10.1093/pubmed/fdr033 21562029

[5] Ding D, Lawson KD, Kolbe-Alexander TL, Finkelstein EA, Katzmarzyk PT, Van Mechelen W, Pratt M. The economic burden of physical inactivity: a global analysis of major non-communicable diseases. Lancet. 2016;388(10051):1311–24. 27475266 10.1016/S0140-6736(16)30383-X

[6] Goodchild M, Nargis N, d’Espaignet ET. Global economic cost of smoking-attributable diseases. Tob Control. 2018;27(1):58–64. 28138063 10.1136/tobaccocontrol-2016-053305 PMC5801657

[7] World Health Organization. WHO recommendations on maternal and newborn care for a positive postnatal experience. 2022. 35467813

[8] Smith D, Lavender T. The maternity experience for women with a body mass index ≥ 30 kg/m2: a meta-synthesis. BJOG: Int J Obstet Gynecol. 2011;118(7):779–89. 10.1111/j.1471-0528.2011.02924.x 21385305

[9] Yelverton CA, Geraghty AA, O’Brien EC, Killeen SL, Horan MK, Donnelly JM, et al. Breastfeeding and maternal eating behaviours are associated with child eating behaviours: Findings from the ROLO kids study. Eur J Clin Nutr. 2020;75(4):670–9. 32999419 10.1038/s41430-020-00764-7 PMC8035071

[10] Olander EK, Darwin ZJ, Atkinson L, Smith DM, Gardner B. Beyond the ‘teachable moment’–A conceptual analysis of women’s perinatal behaviour change. Women Birth. 2016;29(3):e67–71. 26626592 10.1016/j.wombi.2015.11.005

[11] McBride CM, Emmons KM, Lipkus IM. Understanding the potential of teachable moments: the case of smoking cessation. Health Educ Res. 2003;18(2):156–70. 12729175 10.1093/her/18.2.156

[12] Lawson PJ, Flocke SA. Teachable moments for health behavior change: a concept analysis. Patient Educ Couns. 2009;76(1):25–30. 19110395 10.1016/j.pec.2008.11.002 PMC2733160

[13] Flocke SA, Clark E, Antognoli E, Mason MJ, Lawson PJ, Smith S. 10.1016/j.pec.2008.11.002Cohen DJ. Teachable moments for health behavior change and intermediate patient outcomes. Patient Educ Couns. 2014;96(1):43–9. 24856449 10.1016/j.pec.2014.03.014 PMC4427843

[14] Phelan S. Pregnancy: a teachable moment for weight control and obesity prevention. Am J Obstet Gynecol. 2010;202(2):135–e1. 10.1016/j.ajog.2009.06.008 PMC2815033 19683692

[15] Michie S, Van Stralen MM, West R. The behaviour change wheel: a new method for characterising and designing behaviour change interventions. Implement Sci. 2011;6(1):1–12. 21513547 10.1186/1748-5908-6-42 PMC3096582

[16] Rockliffe L, Peters S, Heazell AE, Smith DM. Factors influencing health behaviour change during pregnancy: A systematic review and meta-synthesis. Health Psychol Rev. 2021;15(4):613–32. 34092185 10.1080/17437199.2021.1938632

[17] Rockliffe L, Peters S, Heazell AE, Smith DM. Understanding pregnancy as a teachable moment for behaviour change: a comparison of the COM-B and teachable moments models. Health Psychol Behav Med. 2022;10(1):41–59. 34993005 10.1080/21642850.2021.2014851 PMC8725882

[18] Smith DM, Taylor W, Lavender T. Behaviour change techniques to change the postnatal eating and physical activity behaviours of women who are obese: a qualitative study. BJOG: Int J Obstet Gynecol. 2016;123(2):279–84. 10.1111/1471-0528.13751 26537206

[19] World Health Organization. WHO recommendations on postnatal care of the mother and newborn. 2014. 24624481

[20] Talbot H, Strong E, Peters S, Smith DM. Behaviour change opportunities at mother and baby checks in primary care: a qualitative investigation of the experiences of GPs. Br J Gen Pract. 2018;68(669):e252–9. 29530920 10.3399/bjgp18X695477 PMC5863679

[21] Wolf PhD CP, Jason A, Niederhauser DrPH RN, Marshburn PhD RN, LaVela PhD MP. Defining patient experience. Patient Exp J. 2014;1(1):7–19. 10.35680/2372-0247.1004.

[22] Moher D, Liberati A, Tetzlaff J, Altman DG, PRISMA Group. Preferred reporting items for systematic reviews and meta-analyses: the PRISMA statement. Ann Intern Med. 2009;151(4):264–9. 19622511 10.7326/0003-4819-151-4-200908180-00135

[23] PROSPERO. *Women’s experiences of discussing health behaviours within their perinatal care: a qualitative synthesis* (CRD42022370962). 2022.

[24] Merner B, Schonfeld L, Virgona A, Lowe D, Walsh L, Wardrope C et al. Consumers’ and health providers’ views and perceptions of partnering to improve health services design, delivery and evaluation: a co-produced qualitative evidence synthesis. Cochrane Database Syst Reviews. 2023;3(3):013274. 10.1002/14651858.CD013274.pub2. 10.1002/14651858.CD013274.pub2 PMC10065807 36917094

[25] Cooke A, Smith D, Booth A. Beyond PICO: the SPIDER tool for qualitative evidence synthesis. Qual Health Res. 2012;22(10):1435–43. 22829486 10.1177/1049732312452938

[26] Butler A, Hall H, Copnell B. A guide to writing a qualitative systematic review protocol to enhance evidence-based practice in nursing and health care. Worldviews Evidence‐Based Nurs. 2016;13(3):241–9. 10.1111/wvn.12134 26790142

[27] Rockliffe L. Including non-English language articles in systematic reviews: A reflection on processes for identifying low‐cost sources of translation support. Res Synthesis Methods. 2022;13(1):2–5. 10.1002/jrsm.1508 34169665

[28] Cohen J. A Coefficient of Agreement for Nominal Scales. Educ Psychol Meas. 1960;20(1):37–46.

[29] Critical Appraisal Skills Programme. CASP Qualitative Checklist 2018 [Available from: chrome-extension://efaidnbmnnnibpcajpcglclefindmkaj/https://casp-uk.net/images/checklist/documents/CASP-Qualitative-Studies-Checklist/CASP-Qualitative-Checklist-2018.pdf

[30] Dixon-Woods M, Sutton A, Shaw R, Miller T, Smith J, Young B, et al. Appraising qualitative research for inclusion in systematic reviews: a quantitative and qualitative comparison of three methods. J Health Serv Res Policy. 2007;12(1):42–7. 17244397 10.1258/135581907779497486

[31] Long HA, French DP, Brooks JM. Optimising the value of the critical appraisal skills programme (CASP) tool for quality appraisal in qualitative evidence synthesis. Res Methods Med Health Sci. 2020;1(1):31–42.

[32] Harrisa JL, Bootha A, Cargob M, Hannesc K, Hardend A, Flemminge K, et al. Cochrane Qualitative and Implementation Methods Group guidance series-paper 2: methods for question formulation, searching, and protocol development for qualitative evidence synthesis. J Clin Epidemiol. 2018;97:39–48. 29248725 10.1016/j.jclinepi.2017.10.023

[33] Thomas J, Harden A. Methods for the thematic synthesis of qualitative research in systematic reviews. BMC Med Res Methodol. 2008;8(1):1–10. 18616818 10.1186/1471-2288-8-45 PMC2478656

[34] Clarke V, Braun V. Thematic analysis: a practical guide. Thematic Anal. 2021;1:100.

[35] Candela AG. Exploring the function of member checking. qualitative Rep. 2019;24(3):619–28.

[36] Thomas DR. Feedback from research participants: are member checks useful in qualitative research? Qualitative Res Psychol. 2017;14(1):23–41.

[37] Motulsky SL. Is member checking the gold standard of quality in qualitative research? Qualitative Psychol. 2021;8(3):389.

[38] Garnweidner LM, Pettersen KS, Mosdøl A. Experiences with nutrition-related information during antenatal care of pregnant women of different ethnic backgrounds residing in the area of Oslo. Nor Midwifery. 2013;29(12):e130–7. 23481338 10.1016/j.midw.2012.12.006

[39] Hocking M, O’Callaghan F, Reid N. Women’s experiences of messages relating to alcohol consumption, received during their first antenatal care visit: An interpretative phenomenological analysis. Women Birth. 2020;33(2):e122–8. 30827779 10.1016/j.wombi.2019.02.002

[40] Haugland S, Haug K, Wold B. The pregnant smoker’s experience of ante-natal care—results from a qualitative study. Scand J Prim Health Care. 1996;14(4):216–22. 8956449 10.3109/02813439608997088

[41] Wan CS, Teede H, Nankervis A, Aroni R. Ethnic differences in dietary management of gestational diabetes mellitus: a mixed methods study comparing ethnic Chinese immigrants and Australian women. J Acad Nutr Dietetics. 2020;120(1):86–102. 10.1016/j.jand.2019.08.019 31718911

[42] Baron R, Heesterbeek Q, Manniën J, Hutton EK, Brug J, Westerman MJ. Exploring health education with midwives, as perceived by pregnant women in primary care: A qualitative study in the Netherlands. Midwifery. 2017;46:37–44. 28161688 10.1016/j.midw.2017.01.012

[43] Malik AF, Belizan M, Gutierrez M, Vilajeliu A, Sanclemente LN, Casanova IG, et al. Pregnant women’s perspectives about maternal immunization in Latin America. Vaccine. 2021;39:B44–9. 32972734 10.1016/j.vaccine.2020.09.009

[44] Watson ED, Norris SA, Draper CE, Jones RA, van Poppel MN, Micklesfield LK. Just because you’re pregnant, doesn’t mean you’re sick!’ a qualitative study of beliefs regarding physical activity in black South African women. BMC Pregnancy Childbirth. 2016;16(1):174. 27435173 10.1186/s12884-016-0963-3 PMC4952193

[45] Pledger AB. Exploring the experiences of pregnant women using an NHS stop smoking service: a qualitative study. Perspect public health. 2015;135:138–44. 25925309 10.1177/1757913915577156

[46] Nielsen JH, Olesen CR, Kristiansen TM, Bak CK, Overgaard C. Reasons for women’s non-participation in follow-up screening after gestational diabetes. Women Birth. 2015;28(4):e157–63. 25997731 10.1016/j.wombi.2015.04.006

[47] Lynch MM, Mitchell EW, Williams JL, Brumbaugh K, Jones-Bell M, Pinkney DE, et al. Pregnant and recently pregnant women’s perceptions about influenza a pandemic (H1N1) 2009: implications for public health and provider communication. Matern Child Health J. 2012;16:1657–64. 21822963 10.1007/s10995-011-0865-y

[48] Raymond N, Beer C, Glazebrook C, Sayal K. Pregnant women’s attitudes towards alcohol consumption. BMC Public Health. 2009;9:1–8. 19500375 10.1186/1471-2458-9-175 PMC2701426

[49] Boydell N, Cooper M, Cameron ST, Glasier A, Coutts S, McGuire F, Harden J. Women’s experiences of accessing postpartum intrauterine contraception in a public maternity setting: a qualitative service evaluation. Eur J Contracept Reproductive Health Care. 2020;25(6):465–73. 10.1080/13625187.2020.1815006 32990066

[50] Draffin C, Alderdice FA, McCance DR, Maresh M, Harper R, McSorley O, Holmes VA. Exploring the needs, concerns and knowledge of women diagnosed with gestational diabetes: A qualitative study. Midwifery. 2016;40:141–7. 27553869 10.1016/j.midw.2016.06.019

[51] Chana R, Haith-Cooper M. Diet and physical activity in pregnancy: a study exploring women’s beliefs and behaviours. Br J Midwifery. 2019;27(5):297–304.

[52] Grant A, Morgan M, Mannay D, Gallagher D. Understanding health behaviour in pregnancy and infant feeding intentions in low-income women from the UK through qualitative visual methods and application to the COM-B (Capability, Opportunity, Motivation-Behaviour) model. BMC Pregnancy Childbirth. 2019;19:1–16. 30744581 10.1186/s12884-018-2156-8 PMC6371518

[53] Knight-Agarwal CR, Cubbage R, Sesleja R, Hinder M, Mete R. The nutrition-related information seeking behaviours and attitudes of pregnant women with a high BMI: A qualitative study. Women Birth. 2020;33(3):294–9. 30898337 10.1016/j.wombi.2019.03.005

[54] Atkinson S, McNamara PM. Unconscious collusion: An interpretative phenomenological analysis of the maternity care experiences of women with obesity (BMI ≥ 30 kg/m²). Midwifery. 2017;49:54–64. 28069317 10.1016/j.midw.2016.12.008

[55] Gamble J, Grant J, Tsourtos G. Missed opportunities: a qualitative exploration of the experiences of smoking cessation interventions among socially disadvantaged pregnant women. Women Birth. 2015;28(1):8–15. 25438715 10.1016/j.wombi.2014.11.003

[56] Bianchi CM, Huneau JF, Le Goff G, Verger EO, Mariotti F, Gurviez P. Concerns, attitudes, beliefs and information seeking practices with respect to nutrition-related issues: a qualitative study in French pregnant women. BMC Pregnancy Childbirth. 2016;16:1–14. 27729021 10.1186/s12884-016-1078-6 PMC5059968

[57] Booth K, Sundstrom B, DeMaria AL, Dempsey A. A qualitative analysis of postpartum contraceptive choice. J Communication Healthc. 2018;11(3):215–22.

[58] Dinsdale S, Branch K, Cook L, Shucksmith J. As soon as you’ve had the baby that’s it… a qualitative study of 24 postnatal women on their experience of maternal obesity care pathways. BMC Public Health. 2016;16(1):1–13. 27449265 10.1186/s12889-016-3289-1 PMC4957370

[59] Potgieter F, Kapp P, Coetzee F. Factors influencing post-partum women’s choice of an implantable contraceptive device in a rural district hospital in South Africa. South Afr Family Pract. 2018;60(6):174–80.

[60] Padmanabhan U, Summerbell CD, Heslehurst N. A qualitative study exploring pregnant women’s weight-related attitudes and beliefs in UK: the BLOOM study. BMC Pregnancy Childbirth. 2015;15:1–14. 25895679 10.1186/s12884-015-0522-3 PMC4417529

[61] Ferrari RM, Siega-Riz AM, Evenson KR, Moos MK, Carrier KS. A qualitative study of women’s perceptions of provider advice about diet and physical activity during pregnancy. Patient Educ Couns. 2013;91(3):372–7. 23399436 10.1016/j.pec.2013.01.011 PMC3683874

[62] Bookari K, Yeatman H, Williamson M. Informing nutrition care in the antenatal period: pregnant women’s experiences and need for support. Biomed Res Int. 2017;2017(1):4856527. 28890896 10.1155/2017/4856527 PMC5584352

[63] McKenzie LB, Keim SA, Klebanoff MA. Risk perceptions about cannabis use and receipt of health-related information during pregnancy. Am J Health Promotion. 2022;36(8):1316–25. 10.1177/08901171221099496 PMC9617780 35512115

[64] Lawrence RL, Ward K, Wall CR, Bloomfield FH. New Zealand women’s experiences of managing gestational diabetes through diet: a qualitative study. BMC Pregnancy Childbirth. 2021;21(1):819. 34886814 10.1186/s12884-021-04297-0 PMC8662890

[65] Jarlenski M, Tarr JA, Holland CL, Farrell D, Chang JC. Pregnant women’s access to information about perinatal marijuana use: a qualitative study. Women’s Health Issues. 2016;26(4):452–9. 27131908 10.1016/j.whi.2016.03.010 PMC4958505

[66] Tod AM. Barriers to smoking cessation in pregnancy: a qualitative study. Br J Community Nurs. 2003;8(2):56–64. 12589246 10.12968/bjcn.2003.8.2.11088

[67] Congdon JL, Trope LA, Bruce JS, Chung PJ, Dehlendorf C, Chamberlain LJ. Meeting the needs of postpartum women with and without a recent preterm birth: perceptions of maternal family planning in pediatrics. Matern Child Health J. 2020;24:378–88. 31875305 10.1007/s10995-019-02829-x

[68] Pardell-Dominguez L, Palmieri PA, Dominguez-Cancino KA, Camacho-Rodriguez DE, Edwards JE, Watson J, Leyva-Moral JM. The meaning of postpartum sexual health for women living in Spain: a phenomenological inquiry. BMC Pregnancy Childbirth. 2021;21:1–13. 33509133 10.1186/s12884-021-03578-y PMC7844957

[69] Grenier LN, Atkinson SA, Mottola MF, Wahoush O, Thabane L, Xie F, et al. Be healthy in pregnancy: exploring factors that impact pregnant women’s nutrition and exercise behaviours. Matern Child Nutr. 2021;17(1):e13068. 32705811 10.1111/mcn.13068 PMC7729656

[70] English F, Greyson D. You still have that fear: Policy constraints on informed decision making about legalized cannabis use during pregnancy and lactation. Int J Drug Policy. 2022;106:103774. 35772267 10.1016/j.drugpo.2022.103774

[71] Fernández-Sola C, Huancara-Kana D, Granero-Molina J, Carmona-Samper E, López-Rodríguez MDM, Hernández-Padilla JM. Sexuality throughout all the stages of pregnancy: Experiences of expectant mothers. Acta Paulista de Enfermagem. 2018;31:305–12.

[72] Barbosa-Leiker C, Burduli E, Smith CL, Brooks O, Orr M, Gartstein M. Daily cannabis use during pregnancy and postpartum in a state with legalized recreational cannabis. J Addict Med. 2020;14(6):467–74. 32011411 10.1097/ADM.0000000000000625 PMC7647431

[73] Cunningham J, Endacott R, Gibbons D. Communication with health professionals: The views of pregnant women with a raised BMI. Br J Midwifery. 2018;26(9):598–604.

[74] Knight-Agarwal CR, Williams LT, Davis D, Davey R, Shepherd R, Downing A, Lawson K. The perspectives of obese women receiving antenatal care: A qualitative study of women’s experiences. Women Birth. 2016;29(2):189–95. 26563638 10.1016/j.wombi.2015.10.008

[75] Heslehurst N, Dinsdale S, Brandon H, Johnston C, Summerbell C, Rankin J. Lived experiences of routine antenatal dietetic services among women with obesity: A qualitative phenomenological study. Midwifery. 2017;49:47–53. 27986354 10.1016/j.midw.2016.11.001

[76] Fuss TL, Devera JL, Pierre-Joseph N, Perkins RB. Attitudes and communication preferences for vaccines among pregnant women receiving care at a safety-net hospital. Women’s health issues. 2022;32(1):67–73. 34635378 10.1016/j.whi.2021.09.004

[77] Yuen CY, Dodgson JE, Tarrant M. Perceptions of Hong Kong Chinese women toward influenza vaccination during pregnancy. Vaccine. 2016;34(1):33–40. 26616554 10.1016/j.vaccine.2015.11.032

[78] Martinelli JL, Germano CMR, de Avó LRDS, Fontanella BJB, Melo DG. Alcohol consumption during pregnancy in Brazil: elements of an interpretive approach. Qual Health Res. 2021;31(11):2123–34. 34166121 10.1177/10497323211023443

[79] Sundstrom B, Szabo C, Dempsey A. My body. My choice: A qualitative study of the influence of trust and locus of control on postpartum contraceptive choice. J Health Communication. 2018;23(2):162–9. 29297766 10.1080/10810730.2017.1421728

[80] McTigue G, Swartz A, Brittain K, Rini Z, Colvin CJ, Harrison A et al. Contraceptive trajectories postpartum: a longitudinal qualitative study of women living with HIV in Cape Town, South Africa. Soc Sci Med. 2022;292:114555. 10.1016/j.socscimed.2021.114555. 10.1016/j.socscimed.2021.114555 PMC8748387 34776286

[81] Findley A, Smith DM, Hesketh K, Keyworth C. Exploring womens’ experiences and decision making about physical activity during pregnancy and following birth: A qualitative study. BMC Pregnancy Childbirth. 2020;20:1–10. 10.1186/s12884-019-2707-7 PMC6993510 32000706

[82] Fletcher C, Hoon E, Gialamas A, Dekker G, Lynch J, Smithers L. Isolation, marginalisation and disempowerment–understanding how interactions with health providers can influence smoking cessation in pregnancy. BMC Pregnancy Childbirth. 2022;22(1):396. 35538450 10.1186/s12884-022-04720-0 PMC9086664

[83] Beckham AJ, Urrutia RP, Sahadeo L, Corbie-Smith G, Nicholson W. We know but we don’t really know: Diet, physical activity and cardiovascular disease prevention knowledge and beliefs among underserved pregnant women. Matern Child Health J. 2015;19:1791–801. 25656718 10.1007/s10995-015-1693-2

[84] Sloan M, Campbell KA, Bowker K, Coleman T, Cooper S, Brafman-Price B, Naughton F. Pregnant women’s experiences and views on an opt-out referral pathway to specialist smoking cessation support: A qualitative evaluation. Nicotine Tob Res. 2016;18(5):900–5. 26743356 10.1093/ntr/ntv273 PMC5896838

[85] Stacey T, Samples J, Leadley C, Akester L, Jenney A. I don’t need you to criticise me, I need you to support me’. A qualitative study of women’s experiences of and attitudes to smoking cessation during pregnancy. Women Birth. 2022;35(6):e549–55. 35115246 10.1016/j.wombi.2022.01.010

[86] Jacobson LT, Zackula R, Redmond ML, Duong J, Collins TC. Pioneer baby: suggestions for pre-and postnatal health promotion programs from rural English and Spanish-speaking pregnant and postpartum women. J Behav Med. 2018;41:653–67. 29721813 10.1007/s10865-018-9930-y

[87] Helmersen M, Sørensen M, Lukasse M, Laine HK, Garnweidner-Holme L. Women’s experience with receiving advice on diet and Self-Monitoring of blood glucose for gestational diabetes mellitus: a qualitative study. Scand J Prim Health Care. 2021;39(1):44–50. 33555201 10.1080/02813432.2021.1882077 PMC7971282

[88] Woodruff K, Scott KA, Roberts SC. Pregnant people’s experiences discussing their cannabis use with prenatal care providers in a state with legalized cannabis. Drug Alcohol Depend. 2021;227:108998. 34482037 10.1016/j.drugalcdep.2021.108998

[89] Groves AK, Maman S, Msomi S, Makhanya N, Moodley D. The complexity of consent: women’s experiences testing for HIV at an antenatal clinic in Durban, South Africa. AIDS Care. 2010;22(5):538–44. 20229376 10.1080/09540120903311508 PMC2992088

[90] Arreciado Maranon A, Fernández-Cano MI, Montero‐Pons L, Feijoo‐Cid M, Reyes‐Lacalle A, Cabedo‐Ferreiro RM, et al. Understanding factors that influence the decision to be vaccinated against influenza and pertussis in pregnancy: a qualitative study. J Clin Nurs. 2022;31(11–12):1531–46. 34423873 10.1111/jocn.16006

[91] Rockliffe L, Peters S, Smith DM, Heal C, Heazell AE. Investigating the utility of the COM-B and TM model to explain changes in eating behaviour during pregnancy: A longitudinal cohort study. Br J Health Psychol. 2022;27(3):1077–99. 35297131 10.1111/bjhp.12590 PMC9541598

[92] Public Health England. Achieving behaviour change: a guide for local government and partners. 2020.

[93] Public Health England. Achieving behaviour change: a guide for national government. 2020.

[94] Michie S, Richardson M, Johnston M, Abraham C, Francis J, Hardeman W, et al. The behavior change technique taxonomy (v1) of 93 hierarchically clustered techniques: building an international consensus for the reporting of behavior change interventions. Ann Behav Med. 2013;46(1):81–95. 23512568 10.1007/s12160-013-9486-6

[95] Chisholm A, Hart J, Lam V, Peters S. Current challenges of behavior change talk for medical professionals and trainees. Patient Educ Couns. 2012;87(3):389–94. 22205055 10.1016/j.pec.2011.12.001

[96] Public Health England, England NHS. Health Education England. Making Every Contact Count (MECC): Consensus statement. 2016.

[97] The All-Party Parliamentary Group on Birth Trauma. Listen to Mums: Ending the Postcode Lottery on Perinatal Care. 2024.

[98] Tod AM. Barriers to smoking cessation in pregnancy: a qualitative study. Br J Community Nurs. 2003;8(2):56–64. 12589246 10.12968/bjcn.2003.8.2.11088

[99] Ministry of Public Health. RESPECFUL MATERNITY CARE ORIENTATION PACKAGE for Health Care Providers. Participants Guide.; 2017.

[100] Pollock A, Campbell P, Synnot A, Smith M, Morley R. Patient and public involvement in systematic reviews. GIN Public Toolkit: Patient and Public Involvement in Guidelines; 2021.

[101] National Institute of Health and Care Research. Briefing notes for researchers - public involvement in NHS, health and social care research. 2021.

