# Supplementary material for: Women’s experiences of discussing health behaviours within their maternity care: a systematic review and meta-synthesis
Source: Reprod Health. 2026 May 29;23:152. doi: 10.1186/s12978-026-02368-z (PMC13430855; doi:10.1186/s12978-026-02368-z)
Supplement: Supplementary file 2 — Supplementary Material 2. [file 12978_2026_2368_MOESM2_ESM.pdf]

All behaviours being discussed between women and HCPs.

Alcohol consumption.

Attending screening appointment.

Diet (including vitamin supplement taking).

Exercise.

Medicine adherence (including glycaemic control).

Recreational drug taking.

Sexual health behaviours (Including contraceptive taking, safe sexual practices).

Smoking (including CO testing).

Testing for communicable diseases (including HIV).

Unspecified lifestyle behaviours.

Vaccination taking.
