# Supplementary material for: Women’s experiences of discussing health behaviours within their maternity care: a systematic review and meta-synthesis
Source: Reprod Health. 2026 May 29;23:152. doi: 10.1186/s12978-026-02368-z (PMC13430855; doi:10.1186/s12978-026-02368-z)
Supplement: Supplementary file 3 — Supplementary Material 3. [file 12978_2026_2368_MOESM3_ESM.pdf]

Data extraction tool

**Title:**
**Authors:**
**Year of Publication:**
**Source type:**
**Study type:**
**Study objective:**
**Dates the research was conducted:**
**Participant demographics**<br>• **Number of participants:**<br>• **Ethnicity of participants:**<br>• **Age average or range:**<br>• **1001 days timeframe:**<br>• **Perinatal care provided in what country:**<br>• **Extra perinatal care information:**<br>• **Eligibility criteria:**
**Data collection method:**
**Analysis method:**
**Relevant findings**:
Health behaviours reflected on by women in these findings:
Perinatal care providers brought up by women in these findings:
