# Supplementary material for: Women’s experiences of discussing health behaviours within their maternity care: a systematic review and meta-synthesis
Source: Reprod Health. 2026 May 29;23:152. doi: 10.1186/s12978-026-02368-z (PMC13430855; doi:10.1186/s12978-026-02368-z)
Supplement: Supplementary file 4 — Supplementary Material 4. [file 12978_2026_2368_MOESM4_ESM.pdf]

Search strategies for each database

Maternity & Infant Care Database, MIDIRS (Ovid)

1 (pregnant women or pregnancy).mp. [mp=abstract, heading word, title]

2 antenatal women.mp. [mp=abstract, heading word, title]

3 postnatal women.mp. [mp=abstract, heading word, title]

4 (postpartum women or postpartum period).mp. [mp=abstract, heading word, title]

5 mother.mp. [mp=abstract, heading word, title]

6 1 or 2 or 3 or 4 or 5

7 (health behavio?r* or health or health behavio?r* change or health promotion or lifestyle or life style or behavio?r* change or health-related behavio?r* or health effects).mp. [mp=abstract, heading word, title]

8 interview.mp. [mp=abstract, heading word, title]

9 focus group.mp. [mp=abstract, heading word, title]

10 8 or 9

11 experience.mp. [mp=abstract, heading word, title]

12 expectation.mp. [mp=abstract, heading word, title]

13 perspective.mp. [mp=abstract, heading word, title]

14 perception.mp. [mp=abstract, heading word, title]

15 (emotion or understand* or belie*).mp. [mp=abstract, heading word, title]

16 11 or 12 or 13 or 14 or 15

17 qualitative.mp. [mp=abstract, heading word, title]

18 qualitative research.mp. [mp=abstract, heading word, title]

19 mixed methods.mp. [mp=abstract, heading word, title]

20 17 or 18 or 19

21 6 and 7 and 10 and 16 and 20

MEDLINE (Ovid)

1 pregnant woman.mp. or Pregnant Women/

2 Pregnancy/ or pregnancy.mp.

3 antenatal women.mp.

4 postnatal women.mp.

5 postpartum women.mp. or Postpartum Period/

6 mother.mp. or Mothers/

7 1 or 2 or 3 or 4 or 5 or 6

8 Health Behavior/ or health behavio?r*.mp.

9 Health/ or health.mp.

10 health behavio?r* change.mp.

11 health promotion.mp. or Health Promotion/

12 lifestyle.mp. or Life Style/

13 life style.mp.

14 behavio?r* change.mp.

15 health-related behavio?r*.mp.

16 health effects.mp.

17 8 or 9 or 10 or 11 or 12 or 13 or 14 or 15 or 16

18 Interview/ or interview.mp.

19 focus group.mp. or Focus Groups/

20 18 or 19

21 experience.mp.

22 expectation.mp.

23 perspective.mp.

24 perception.mp. or Perception/

25 emotion.mp. or Emotions/

26 understand*.mp.

27 belie*.mp.

28 21 or 22 or 23 or 24 or 25 or 26 or 27

29 qualitative.mp.

30 qualitative research.mp. or Qualitative Research/

31 mixed methods.mp.

32 29 or 30 or 31

33 7 and 17 and 20 and 28 and 32

PsycINFO (Ovid)

1 pregnant women.mp.

2 exp Pregnancy/ or pregnancy.mp.

3 antenatal women.mp.

4 exp Postnatal Period/ or postnatal women.mp.

5 postpartum women.mp.

6 mother.mp. or exp Expectant Mothers/ or exp Mothers/

7 1 or 2 or 3 or 4 or 5 or 6

8 exp Health Behavior/ or health beahvio?r*.mp.

9 health.mp. or exp Health/

10 health behavio?r* change.mp.

11 health promotion.mp. or exp Health Promotion/

12 exp Lifestyle Changes/ or exp Lifestyle/ or lifestyle.mp.

13 life style.mp.

14 exp Behavior Change/ or behavio?r* change.mp.

15 health-related behavio?r*.mp.

16 health effects.mp.

17 8 or 9 or 10 or 11 or 12 or 13 or 14 or 15 or 16

18 exp Interviews/ or interview.mp.

19 exp Focus Group/ or focus group.mp.

20 18 or 19

21 experience.mp.

22 exp Expectations/ or exp Parental Expectations/ or expectation.mp.

23 perspective.mp.

24 perception.mp. or exp Perception/

25 emotion.mp. or exp Emotions/

26 understand*.mp.

27 belie*.mp.

28 21 or 22 or 23 or 24 or 25 or 26 or 27

29 exp Qualitative Methods/ or qualitative.mp.

30 qualitative research.mp.

31 mixed methods.mp. or exp Mixed Methods Research/

32 29 or 30 or 31

33 7 and 17 and 20 and 28 and 32

Cumulative Index to Nursing and Allied Health Literature – Plus, CINHAL-P (EBSCOhost)

S1 pregnant women OR pregnancy OR antenatal women OR postnatal women OR postpartum women OR postpartum period OR mother

S2 health behavio?r* OR health OR health behavio?r* change OR health promotion OR lifestyle OR life style OR behavio?r* change OR health-related behavio?r* OR health effects

S3 interview OR focus group

S4 experience OR expectations OR perspective OR perception OR emotion OR understand* OR belie*

S5 qualitative OR qualitative research OR mixed methods

S6 S1 AND S2 AND S3 AND S4 AND S5
